# Dual Visible and NIR Emission, Mechanoluminescence, and Magnetic Properties of PPh_4_[*Ln*L_4_] Chelates with Diphenyl-N-Benzoylamidophosphate

**DOI:** 10.3390/molecules30061245

**Published:** 2025-03-10

**Authors:** Nataliia Kariaka, Dmytro Panasiuk, Viktor Trush, Sergii Smola, Nataliia Rusakova, Viktoriya Dyakonenko, Svitlana Shishkina, Aneta Lipa, Alina Bienko, Justyna Nasalska, Paula Gawryszewska, Volodymyr Amirkhanov

**Affiliations:** 1 Department of Chemistry, Taras Shevchenko National University of Kyiv, Volodymyrska Str. 64, 01601 Kyiv, Ukraine; dpan9294@gmail.com (D.P.); viktor.trush@knu.ua (V.T.); v_amirkhanov@knu.ua (V.A.); 2 A.V. Bogatsky Physicochemical Institute, National Academy of Sciences of Ukraine, 86 Lustdorfska doroga, 65080 Odesa, Ukraine; sssmola@gmail.com (S.S.); natavrusakova@gmail.com (N.R.); 3 SSI “Institute for Single Crystals”, National Academy of Sciences of Ukraine Nauky Ave. 60, 61001 Kharkiv, Ukraine; dyakvik@gmail.com (V.D.); sveta@xray.isc.kharkov.com (S.S.); 4 Institute of Organic Chemistry, National Academy of Sciences of Ukraine, 5, Academician Str., 02660 Kyiv, Ukraine; 5 Faculty of Chemistry, University of Wroclaw, 14 F. Joliot-Curie Str., 50-383 Wroclaw, Poland; aneta.lipa@uwr.edu.pl (A.L.); alina.bienko@uwr.edu.pl (A.B.); justyna.nasalska@uwr.edu.pl (J.N.)

**Keywords:** crystal structure, carbacylamidophosphate, luminescence, single-molecule magnet, coordination compounds, mechanoluminescence, *Nd*, *Sm*, *Dy*, *Tm*

## Abstract

The design, synthesis, and study of lanthanide coordination compounds with luminescent and magnetic properties attractive in modern technologies is still a pressing and challenging task. In the present work, a series of coordination compounds of tetrakis-carbacylamidophosphate **PPh_4_[*Ln*L_4_]** (where HL = diphenyl-N-benzoylamidophosphate) with several lanthanide ions such as *Nd^III^*, *Sm^III^*, *Dy^III^*, and *Tm^III^* was prepared and studied by X-ray analysis and luminescence spectroscopy at 293 and 77 K, as well as by magnetic measurements. Coordination compounds are not isostructural, but the type of coordination is the same. All of them have intense sensitized emission. **PPh_4_[*Sm*L_4_]**, **PPh_4_[*Dy*L_4_]**, and **PPh_4_[*Tm*L_4_]** chelates are characterized by dual visible and infrared emission and mechanoluminescence. In addition, **PPh_4_[*Dy*L_4_]** has multifunctional properties such as Vis and NIR emissions, brilliant mechanoluminescence and single-ion molecular magnet (SIM) properties. This type of compound holds great promise in multifunctional magnetic radiation converters.

## 1. Introduction

The luminescence of lanthanides is highly valuable due to their pure color and long lifetime and has been employed in a variety of areas including lighting, displays, telecommunications, lasers, security inks, barcoding, pressure sensors, molecular thermometers, immunoassays, and bioimaging [1]. The luminescence of *Eu^III^* and *Tb^III^* ions, which possess the longest lifetime, is most studied in bioimaging and electroluminescence devices [2,3,4]. It is worth noting that the luminescence of other lanthanides is less reported; however, it is not less valuable and may find practical applications. For example, *Sm^III^* luminescence is studied because of its application in OLEDs, laser-active materials, solar concentrators, agricultural films, and anticounterfeiting materials [5,6]. *Dy^III^* is considered a source of white light for organic light-emitting device (OLED) technology [7,8], and its near-infrared emission is of interest in the context of optical amplifiers [9]. The luminescent *Tm^III^* compounds are studied because of their potential application in optical communication systems, in OLEDs, and as 0.82, 1.5, 1.8, and 2.34 µm lasers for optical sensing, metrology, and medicine [10,11]. The *Nd^III^* ion’s luminescence in the NIR region is promising for optical communications and biological and sensor applications [12,13].

Complexes of lanthanide ions are also considered the basis for the creation of new magnetic materials. Due to the high magnetic anisotropy of some of *Ln^III^* ions (*Dy^III^*, *Ho^III^*, *Er^III^*), their complexes can have the properties of single-molecule magnets (SMMs) [14,15] with potential applications in high-density information storage and quantum processing [16,17]. Among lanthanide ions, the largest number of SMMs has been found among *Dy^III^* compounds [18,19]. It is worth noting that despite numerous publications on SMM behavior, there are still few reports on *Dy^III^* bifunctional magneto-luminescent compounds due to the location of the emitting dysprosium level at relatively high energy. Thus, the search for ligands, which can serve as efficient sensitizers of *Dy^III^* luminescence and provide a suitable coordination environment for SMM behavior, is an important task in the coordination chemistry of lanthanides.

**Scheme 1:**
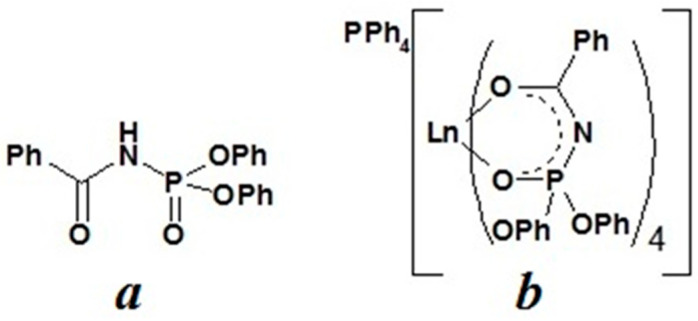
A schematic representation of HL (**a**) and **PPh_4_[*Ln*L_4_]** compounds (**b**).

Recently, we have reported highly luminescent and triboluminescent coordination compounds of *Eu^III^* and *Tb^III^* ions with carbacylamidophosphate ligand–diphenyl-N-benzoylamidophosphate (HL) of the general formula **PPh_4_[*Ln*L_4_]** (Scheme 1) [20]. Considering the good stability of complexes of this type during rather long-term storage and the suitability of the ligand’s lowest triplet state for the sensitization of lanthanide luminescence, we have extended studies of tetrakis-complexes **PPh_4_[*Ln*L_4_]** by the introduction of other lanthanide ions, such as *Nd^III^*, *Sm^III^*, *Dy^III^*, and *Tm^III^.* The complexes were structurally characterized; their luminescent properties were investigated; and magnetic studies of **PPh_4_[*Dy*L_4_]** were conducted.

## 2. Results and Discussion

### 2.1. X-Ray Analysis

**PPh_4_[*Nd*L_4_]**, **PPh_4_[*Sm*L_4_]**, **PPh_4_[*Dy*L_4_]**, and **PPh_4_[*Tm*L_4_]** coordination compounds have similar molecular structures, but **PPh_4_[*Nd*L_4_]** and **PPh_4_[*Tm*L_4_]** crystallize in the tetragonal space group I$\bar{4}$, while the **PPh_4_[*Sm*L_4_]** and **PPh_4_[*Dy*L_4_]** compounds crystallize in the monoclinic space group *I*2. It was found that the *Nd^III^* and *Tm^III^* complexes are isostructural to the previously described gadolinium compound **PPh_4_[*Gd*L_4_]** [20]. There are two complexes of **PPh_4_[*Nd*L_4_]** in the asymmetric part of the unit cell. The first complex exists in a general position, and the Nd2 atom of the anion and P6 atom of the cation of the second complex are located in a special position on the four-order axis. The **PPh_4_[*Sm*L_4_]** and **PPh_4_[*Dy*L_4_]** compounds are isostructural with each other; however, they are not isostructural with the **PPh_4_[*Nd*/*Gd*L_4_]** complexes (Table S1). In the asymmetric part of the **PPh_4_[*Sm*/*Dy*L_4_]** unit cell, there are three **[*Sm/Dy*L_4_]**^−^ anions and a PPh_4_^+^ cation with one anion and one cation molecule in a special position on the two-order axis.

**Figure 1:**
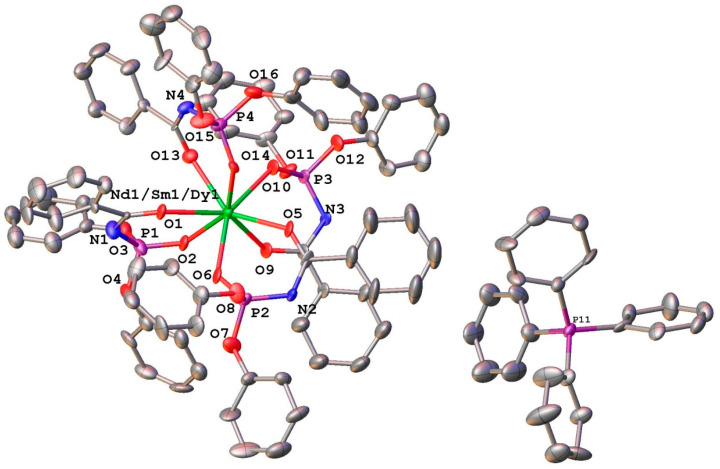
The molecular structures of one of the [*Nd*/*Sm*/*Dy*(L)_4_]^−^ anions (**left**) and PPh_4_^+^ cations (**right**) for **PPh_4_[*Ln*L_4_]** (*Ln* = *Nd*, *Sm*, *Dy*). The hydrogen atoms are omitted for clarity. C atoms are coloured in grey, O atoms—in red, N atoms in blue, P atoms in violet and *Ln*—in green.

The lanthanide ion (*Ln* = *Nd*, *Sm*, *Dy*) in **[*Ln*L_4_]**^−^ anions is coordinated by eight oxygen atoms of four ligands (Figure 1). The CAPh ligands are coordinated to the Ln^3+^ ion in a bidentate chelating manner through oxygen atoms of phosphoryl and carbonyl groups. The values of the *Ln*–O bond lengths in the lanthanide coordination environment of **[*Ln*L_4_**]^−^ anions are within 2.303(10)–2.471(6) Å (Table 1). The *Ln*–O bonds’ lengths in the obtained complexes are comparable with those of reported carbacylamidophosphate-based neodymium, samarium, and dysprosium tetrakis-complexes [21,22].

The lanthanides’ coordination polyhedron geometry in the obtained complexes was analyzed using SHAPE 2.1. software (Table 1 and Table S2) [23]. The lanthanides have different coordination polyhedra in the **[LnL_4_]**^−^ anions of crystallographically independent complexes. The Nd2 atom coordination polyhedron was determined to be a nearly perfect triangular dodecahedron, while the polyhedrons of the other lanthanide atoms were interpreted as intermediate between several geometries such as square antiprism (*D*_4d_), triangular dodecahedron (*D*_2d_), and biaugmented trigonal prism (*C*_2v_) with different degrees of distortion. Based on the smallest value of convergence factors, polyhedron types can finally be assigned, as shown in Table S2.

The coordination of lanthanide ions by CAPh ligands in all **[*Ln*L_4_]**^−^ (*Ln* = *Nd*, *Sm*, *Dy*) anions leads to the formation of six-membered metal cycles Ln–O–C–N–P–O. These cycles adopt twist-boat conformations (Table S3). All conformational characteristics for metal cycles in all symmetrically independent anionic molecules, including deviations from the mean square plane, are given in Table S3.

In the crystal phase, the anions and cations of **PPh_4_[*Ln*L_4_]** (*Ln* = *Nd*, *Sm*, *Dy*) are linked by numerous intermolecular weak interactions, H•••H, H•••C, C•••C, and H•••O, and form a 3D network of molecules (see Table S4).

### 2.2. Photoluminescence Analysis

Previously, diphenyl-N-benzoylamidophosphate was reported to be a very good sensitizer of *Eu^III^* and *Tb^III^* luminescence [20]. The lowest ligand triplet state (LLTS) in complexes of **PPh_4_[*Ln*L_4_]** was shown to be 26,954 cm^−1^ (as a zero-phonon line). This value is high enough to allow not only the sensitization of *Eu^III^* and *Tb^III^* luminescence but also the emission of lanthanides with a higher energy of emissive levels, i.e., *Dy^III^* and *Tm^III^*. The LLTS in **PPh_4_[*Ln*L_4_]** is higher than the emissive levels of the *Nd^III^*, *Sm^III^*, *Dy^III^*, and *Tm^III^* ions (Figure S3). The energy gaps between the LLTS and emissive levels of the mentioned lanthanides in the considered complexes are close to 15,450, 9050, 5850, and 5600 cm^−1^ (^1^G_4_-emitting level), respectively. Although in some publications the authors consider the transfer of excitation energy from the ligand directly to the *Ln^III^*-emitting level, it is known that the transfer can occur to other Ln excited levels. This process depends on many factors, such as the donor–acceptor distance, the mutual spatial position of the donor and acceptor, the rate of competing processes, and the mechanism of energy transfer [24,25,26], and not just on the energy gap between the donor (ligand) and acceptor (*Ln^III^* ion) states. In [25], it was proved for the first time, based on experimental results and theoretical calculations, that energy transfer from the ligand singlet state (S_1_) occurs and can be a dominant process. In contrast, using fs transient absorption, it was shown experimentally that energy transfer from the ligand triplet state (T_1_) to the nearest excited level of the *Ln^III^* ion is dominant for a series of lanthanide compounds [27].

**Figure 2:**
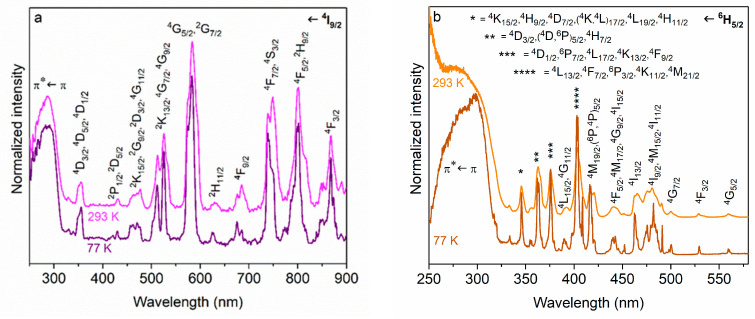

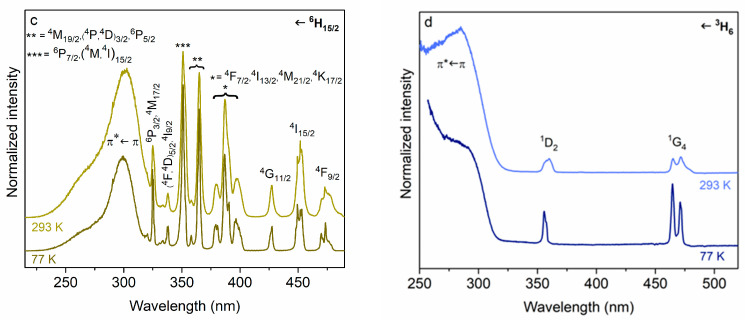
Luminescence excitation spectra of (**a**) **PPh_4_[*Nd*L_4_]** λ_em_ = 1055.4 nm; (**b**) **PPh_4_[*Sm*L_4_]** λ_em_ = 637 nm; (**c**) **PPh_4_[*Dy*L_4_]** λ_em_ = 574 nm; and (**d**) **PPh_4_[*Tm*L_4_]** λ_em_ = 776 nm in single-crystal form at 293 and 77 K.

Luminescence excitation spectra of all studied compounds are presented in Figure 2. On the spectra, in the spectral range of 250–320 nm, a band corresponding to a π* ← π-type ligand absorption is present. In addition, bands of small half-width corresponding to 4f* ← 4f-type transitions are visible on the excitation spectra. Energy transfer requires spectral overlap between donor emission and acceptor absorption. It can be seen that all the studied *Ln^III^* ions have numerous absorption transitions in the ligand phosphorescence spectral range (ligand phosphorescence in the range of about 370–550 nm [20]). The *Ln^III^* transitions are described based on data developed by Carnall [28]. The highest relative ratio of the intensity of ligand absorption to 4f* ← 4f transitions is observed for *Sm^III^* and *Tm^III^*, indicating the occurrence of the most efficient energy transfer for these compounds (Figure 2b,d). Taking into account selection rules [29], the intramolecular energy transfer from T_1_ can occur to several *Sm^III^* levels and to the ^1^G_4_ *Tm^III^* level by multipolar (dipole–2^λ^ pole and dipole–dipole) mechanisms (|*J* − *J*’| ≤λ≤ |*J* + *J*’|, *J*’ = *J* = 0 are excluded). In addition, energy transfer for **PPh_4_[*Sm*L_4_]** can also be mediated through an exchange mechanism ($\left|J-J^{\prime }\right|=0\;or\;1,$ *J*’ = *J* = 0 are excluded in the case of ligand-to-metal energy transfer). It should be emphasized that intramolecular energy transfer from S_1_ to ^1^D_2_ *Tm^III^* cannot be excluded. The **PPh_4_[*Tm*L_4_]** excitation spectra presented in Figure 2d were the result of monitoring at 776 nm emission. Other excitation spectra were obtained from the monitoring of ^1^G_4_ → ^3^H_6_ emission (λ_em_ = 472.5 nm (293 K); λ_em_ = 480.2 nm (77 K)). An additional broad band in the 340–420 nm range appeared (Figure S4). Identical results were obtained using different equipment and applying appropriate filters. The intensity of the broad band increases at 77 K. This result is unexpected and requires further study. The ligand absorption bands’ positions nearly coincide in the excitation and diffuse reflection spectra of the compounds (Figure S5). As can be seen in Figure S5, the profile of the reflectance spectra is the same for coordination compounds **PPh_4_[*Gd*L_4_]** and **PPh_4_[*Tm*L_4_]**.

In the case of **PPh_4_[*Nd*L_4_]** and **PPh_4_[*Dy*L_4_]**, efficient ligand-to-*Ln^III^* energy transfer was also observed (see Figure 2a,c), which may be mediated by both multipolar and exchange mechanisms via the T_1_ state and possibly S_1_ as well. It is important to note that the exchange mechanism requires overlap between the donor and acceptor orbitals, so the donor–acceptor distance is crucial in this case. In addition, we would like to point out that the amount of sample and type of holder can affect the values of the relative intensity ratios of ligand-to-*Ln^III^* ion transitions or the shape of the ligand band in the excitation spectrum. This is common knowledge, but sometimes, certain technical aspects are not taken into account. These aspects are particularly important when the ligand is strongly absorbed, in which case a thin layer of the compound should be used to measure excitation spectra. The saturation effect can be manifested by various phenomena [30]. In our case, we noticed slight differences in the intensity ratios of the ligand/*Ln* bands only for the **PPh_4_[*Nd*L_4_]** compound, as shown in Figure S6.

At 77 K, a narrowing of *Ln^III^* ion bands and component formation are observed compared to at room temperature, which is due to the partial depopulation of the ground-state Stark sublevels. However, it is difficult to determine the splitting of the excited *Ln^III^* levels by the crystal field due to the population of some of the ground-state Stark sublevels at 77 K, as well as the presence of *Ln^III^* ions in the crystal structure in two (**PPh_4_[*Nd*L_4_]**) and three (**PPh_4_[*Sm*L_4_]**, **PPh_4_[*Dy*L_4_]**, **PPh_4_[*Tm*L_4_]**) non-equivalent crystallographic positions.

**Figure 3:**
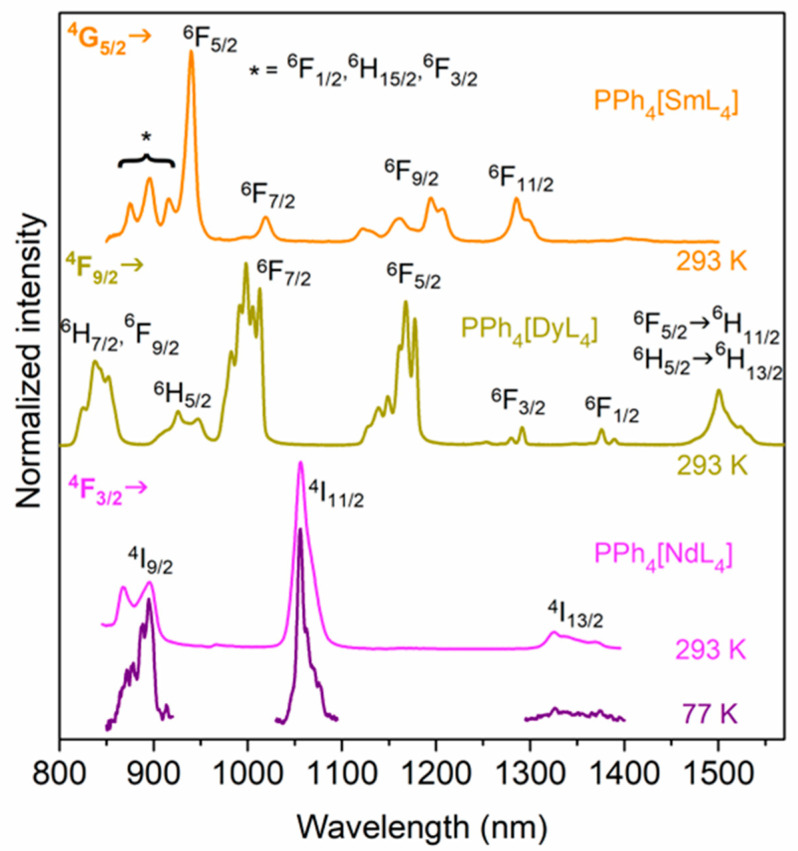
Luminescence spectra of **PPh_4_[*Nd*L_4_]**, **PPh_4_[*Sm*L_4_]**, and **PPh_4_[*Dy*L_4_]** (NIR emission) in single-crystal form at 293-77 K and 293 K, respectively.

**Figure 4:**
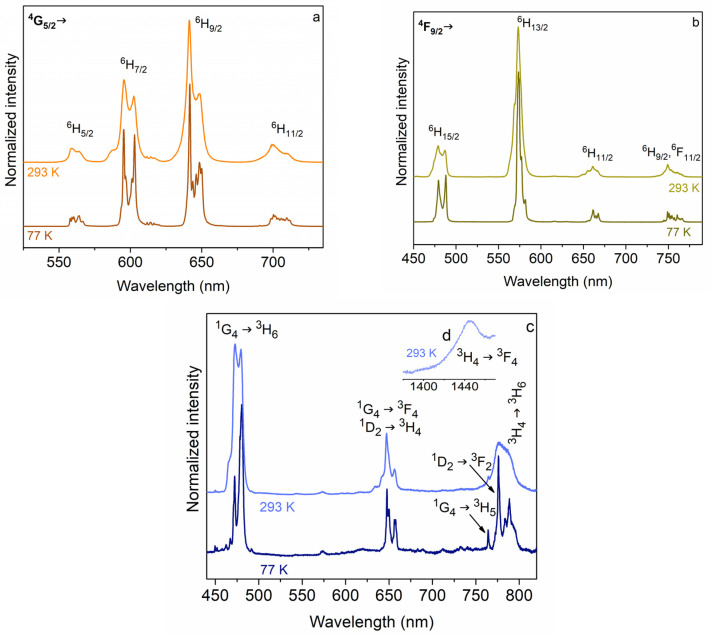
Luminescence spectra of (**a**) **PPh_4_[*Sm*L_4_]**, (**b**) **PPh_4_[*Dy*L_4_]**, and (**c**) **PPh_4_[*Tm*L_4_]** in the form of single crystals.

All compounds show emission after ligand excitation with 285–300 nm radiation at room temperature and 77 K. Coordination compounds **PPh_4_[*Sm*L_4_]**, **PPh_4_[*Dy*L_4_]**, and **PPh_4_[*Tm*L_4_]** demonstrated dual NIR (Figure 3) and visible (Figure 4) luminescence. The neodymium complex **PPh_4_[*Nd*L_4_]** has shown f-f emission typical of *Nd^III^* ions in the NIR region upon excitation of the sample by UV light of a 280 nm wavelength (Figure 3).

The three observed luminescence bands for **PPh_4_[*Nd*L_4_]** have been assigned to the ^4^F_3/2_ → ^4^I_9/2_, ^4^F_3/2_ → ^4^I_11/2_, and ^4^F_3/2_ → ^4^I_13/2_ transitions. The spectrum is dominated by the ^4^F_3/2_ → ^4^I_11/2_ transition band with a maximum at about 1055 nm. Lowering the temperature causes the narrowing of the bands on the higher energy side as a result of the partial depopulation of the higher Stark components of the ^4^F_3/2_ level. This is particularly visible for the ^4^F_3/2_ → ^4^I_11/2_ transition. No separation of emission lines into individual Stark components of the ^4^I_J_ levels is observed due to the lack of complete depopulation of the higher Stark sublevels of the ^4^F_3/2_ level at 77 K as well as the presence of two **[*Nd*L_4_]**^−^ anionic complexes in the **PPh_4_[*Nd*L_4_]** crystal structure.

A large contribution of *Sm^III^* ion luminescence output is situated in the near-infrared region. The emitting level for the near-infrared luminescence of the *Sm^III^* ion is the same as for visible emission—^4^G_5/2_. The NIR luminescence bands of **PPh_4_[*Sm*L_4_]** and their assignments are given in Figure 3. The band of the transition ^4^G_5/2_ → ^6^F_5/2_ with a maximum at 940 nm is dominant in the NIR luminescence spectrum, which is typical for CAPh-based *Sm^III^* coordination compounds.

The *Dy^III^* ion has several important infrared transitions in the near-IR region, which are of interest in optical communications [9]; 0.8, 1.31, and 1.55 μm are, respectively, the first, second, and third telecommunications windows. To our knowledge, there are only two reports on the NIR luminescence of carbacylamidophosphate-based *Dy^III^* complexes [22,31]. The near-IR luminescence of **PPh_4_[*Dy*L_4_]** was registered in the region of 800–1600 nm (see Figure 3). There are several bands with some crystal-field fine structures in the spectrum. The bands of transitions ^4^F_9/2_ → ^6^F_7/2_ (970–1025 nm) and ^4^F_9/2_ → ^6^F_5/2_ (1130–1205 nm) dominate in the obtained spectrum of **PPh_4_[*Dy*L_4_]**. The other less intense bands in the NIR luminescence spectrum of **PPh_4_[*Dy*L_4_]** have been assigned to transitions ^4^F_9/2_ → ^6^H_7/2_,^6^F_9/2_ (810–877 nm) and ^4^F_9/2_ → ^6^H_5/2_ (900–960 nm). We have assigned the bands occurring in the 1230–1400 nm range to the transitions ^4^F_9/2_ → ^6^F_3/2_ (1230–1300 nm) and ^4^F_9/2_ → ^6^F_1/2_ (1330–1400 nm). Based on experimental data, the calculated energy gaps between the ^6^F_1/2_–^6^F_3/2_ and ^6^F_1/2_–^6^F_7/2_, ^6^F_3/2_–^6^F_7/2_ levels are about 500 cm^−1^ and 2730, 2260 cm^−1^, respectively, which is in agreement with Carnall’s results (540 cm^−1^, 2724 cm^−1^, 2184 cm^−1^) [28]. However, it should be noted that in the literature on *Dy^III^*-doped inorganic matrices, the broad emission band in the range of 1250–1400 cm^−1^ is described as a ^6^F_11/2_,^6^H_9/2_ → ^6^H_15/2_ transition. We cannot exclude the contribution of this transition to the observed emission, although the transition suffers from multiphonon relaxation, since the energy gap between the ^6^F_11/2_, ^6^H_9/2_ level and its lower energy level (^6^H_11/2_) is only about 1830 cm^−1^. It is interesting to note the presence of a relatively intense band (1460–1550 nm) in the range of the third telecommunication window. It should be remembered that the studied compounds have relatively high-energy vibrations (see the Experimental Section) associated with organic ligands. The presence of this band is very rare for *Dy^III^* chelates. This band corresponds to the transitions ^6^F_5/2_ → ^6^H_11/2_ and ^6^H_5/2_ → ^6^H_13/2_.

The *Tm^III^* ion f-f transitions in the NIR region occur from its second resonance level ^3^H_4_ (~12,700 cm^−1^) [28]. For the **PPh_4_[*Tm*L_4_]** compound, only one luminescence band with a maximum at 1445 nm is observed in the NIR region (an insert on Figure 4c). The band was assigned to the ^3^H_4_ → ^3^F_4_ transition. It is of interest in optical communication systems [10]. In this case, it should be emphasized again that this emission is rare for the *Tm^III^* compound with organic ligands, because the energy gap between the ^3^H_4_ level and the adjacent level is small and amounts to about 4400 cm^−1^.

In the region of visible light, four narrow bands with maxima at 560, 596, 642, and 700 nm are observed for the **PPh_4_[*Sm*L_4_]** compound (Figure 4a). These bands have been assigned to ^4^G_5/2_ → ^6^H_J_ transitions (*J* = 5/2, 7/2, 9/2, and 11/2, respectively). The band of the ^4^G_5/2_ → ^6^H_9/2_ transition is dominant in the spectrum, unlike *Sm^III^* tetrakis-complexes with dimethyl-N-benzoylamidophospahate [32]. The value of the intensity parameter η_Sm_, which is the ratio of the hypersensitive (electric dipole) ^4^G_5/2_ → ^6^H_9/2_ and magnetic dipole ^4^G_5/2_ → ^6^H_5/2_ transitions [33], for the spectrum of **PPh_4_[*Sm*L_4_]** equals 8.1. This is higher compared to crystalline dimethyl-N-benzoylamidophospahate-based *Sm^III^ tetrakis*-complexes (4.6–5.5), but comparable with their luminescence spectra in solution (8.4–8.6) [32]. The CIE coordinates x, y for **PPh_4_[*Sm*L_4_]** equal 0.622, 0.377, which correspond to red emission (Figure S7). The ^4^G_5/2_ emission lifetime of **PPh_4_[*Sm*L_4_]** was measured at 293 and 77 K by monitoring the intensity of the ^4^G_5/2_ → ^6^H_9/2_ transition and excitation of crystal samples to the 1π* level of the ligand. The luminescence decay curves have been fitted by a single exponential function yielding a value of 224 (293 K) and 255 μs (77 K). The obtained value is the highest among those known for CAPh-based samarium complexes [32,34].

The luminescence spectra of the **PPh_4_[*Dy*L_4_]** compound also contain four narrow bands in the visible light region (Figure 4b). These bands have maxima at 479, 574, 662, and 749 nm, the first three of which correspond to ^4^F_9/2_ → ^6^H_J_ transitions (*J* = 15/2, 13/2, 11/2), while the last one corresponds to ^4^F_9/2_ → ^6^H_9/2_ + ^6^F_11/2_ transitions. The band of the hypersensitive transition ^4^F_9/2_ → ^6^H_13/2_ (574 nm), which corresponds to yellow emission, is stronger than that for blue emission (479 nm). The blue/yellow intensity ratio (ratio of integral intensities of the bands (^4^F_9/2_ → ^6^H_15/2_)/(^4^F_9/2_ → ^6^H_13/2_)) equals 0.36, which is comparable with values reported for CAPh-based dysprosium coordination compounds (0.30–0.47) [31]. The bands of transitions ^4^F_9/2_ → ^6^H_11/2_ (662 nm) and ^4^F_9/2_ → ^6^H_9/2_ + ^6^F_11/2_ (749 nm) are typically of small intensity. The CIE coordinates x, y for **PPh_4_[*Dy*L_4_]** equal 0.41, 0.457, which correspond to yellow emission. The emission lifetime of **PPh_4_[*Dy*L_4_]** is dependent on the temperature and equals 136 μs at room temperature and 171 μs at 77 K.

The *Tm^III^* ion has the highest energy of the emissive level among the lanthanides emitting in the visible region. Thus, it requires ligands with a high energy of the LLTS to achieve the sensitization effect. This is one of the reasons why the luminescence of *Tm^III^* complexes is rather rarely reported. The emission spectrum of **PPh_4_[*Tm*L_4_]** in the visible region shows three bands with maxima at 479, 648, and 780 nm (see Figure 4c). The bands were assigned to the transitions ^1^G_4_ → ^3^H_6_, ^1^G_4_ → ^3^F_4_/^1^D_2_ → ^3^H_4_, and ^1^G_4_ → ^3^H_5_ + ^1^D_2_ → ^3^F_2_ + ^3^H_4_ → ^3^H_6_, respectively. The dominating band is for the ^1^G_4_ → ^3^H_6_ transition, which corresponds to blue emission. Emission from the ^1^D_2_ level, which is located at energy higher than the LLTS, may indicate the presence of intramolecular excitation energy transfer from the ligand singlet state to the ^1^D_2_ level, although the ^3^P_6_ level of *Tm^III^* is also excited by 280 nm radiation. The CIE coordinates x, y for **PPh_4_[*Tm*L_4_]** equal 0.209, 0.164, which correspond to blue–violet emission (Figure S7).

No fluorescence or phosphorescence of the ligands was observed in the luminescence spectra for all investigated compounds. This confirms the efficient excitation energy transfer from the ligands to the lanthanides.

### 2.3. Mechanoluminescence Analysis

**Figure 5:**
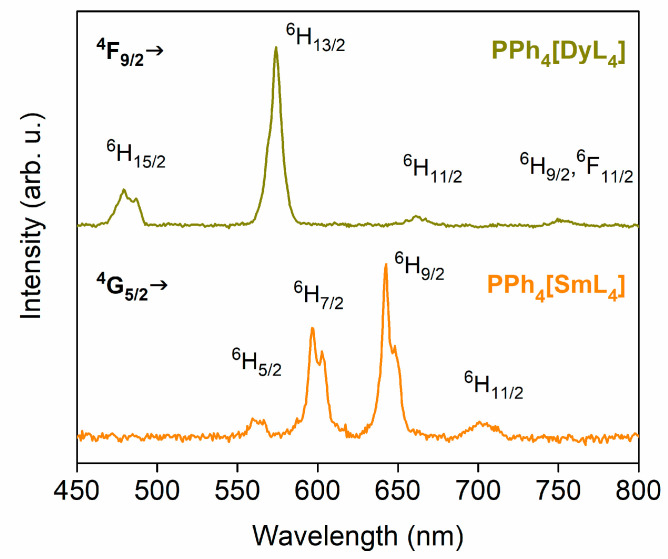
Mechanoluminescence of single crystals of **PPh_4_[*Sm*L_4_]** and **PPh_4_[*Dy*L_4_].**

Analogous to **PPh_4_[*Eu*L_4_]** and **PPh_4_[*Tb*L_4_]** compounds [20], **PPh_4_[*Dy*L_4_]** exhibits intense mechanoluminescence, which is highly visible to the naked eye, as can be seen in the attached video. Flashes of yellow mechanoluminescence appear even when the crystals are lightly rubbed and persist throughout their crushing. The **PPh_4_[*Dy*L_4_]** compound crystallizes in a piezoelectric group, and there are many non-covalent intra- and intermolecular interactions in the structure, so that as a result of rubbing and crushing, the surfaces of the crystals are highly charged, which generates an intense electric field, electrons, and positive ions. These results in the direct population of the excited state of *Dy^III^* ^4^F_9/2_, followed by efficient ^4^F_9/2_ → ^6^H_J_ transitions. The mechanoluminescence spectrum profile (see Figure 5) is similar to the photoluminescence one in the region of ^4^F_9/2_ → ^6^H_15/2_, ^4^F_9/2_ → ^6^H_13/2_, ^4^F_9/2_ → ^6^H_11/2_, and ^4^F_9/2_ → ^6^H_9/2_,^6^F_11/2_ transitions. *Dy^III^* emission in the NIR region caused by rubbing is not observed. Considering the definitions used in the context of mechanoluminescence related to its mechanisms [35], it is likely that the observed mechanoluminescence can be classified as triboluminescence [35]. It should be noted that to our knowledge, only four papers on the mechanoluminescence of *Dy* [36,37,38,39] for *Dy^III^* coordination compounds have been published to date. 

In Figure 5, the mechanoluminescence of **PPh_4_[*Sm*L_4_]** is also visible. It is noteworthy that the weak mechanoluminescence of **PPh_4_[*Tm*L_4_]** can be visually observed, but unfortunately, its intensity is too low for our equipment to record. This is the first report, to our knowledge, of the mechanoluminescence of a *Tm^III^* coordination compound.

### 2.4. Magnetic Properties

#### 2.4.1. DC Magnetic Measurements

The susceptibility data were corrected to the background signals and underlying diamagnetism [40] and transformed to the effective magnetic moment. The magnetization data were collected at low temperatures *T* = 2.0 until *B*_max_ = 7.0 T. No remnant magnetization has been detected.

**Figure 6:**
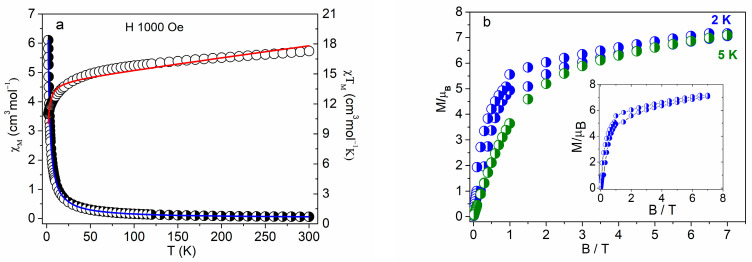

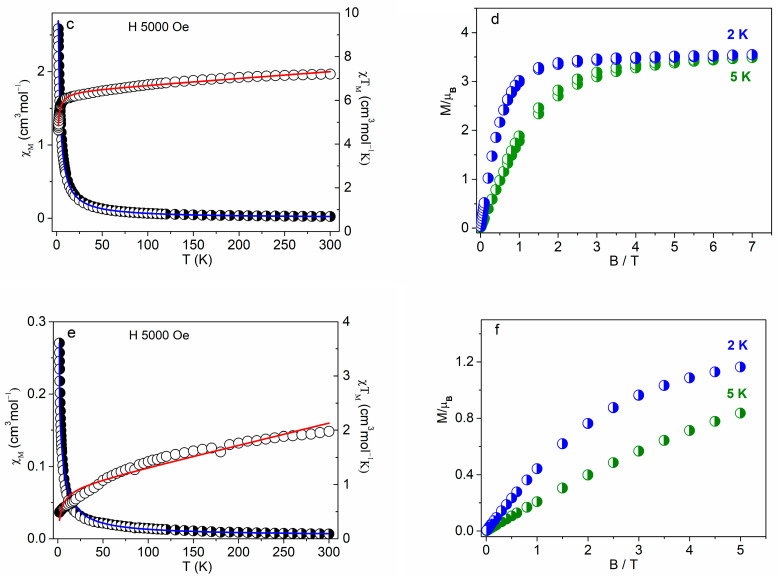
DC magnetic data for **PPh_4_[*Dy*L_4_]** (**a**,**b**), **PPh_4_[*Tm*L_4_]** (**c**,**d**), and **PPh_4_[*Nd*L_4_]** (**e**,**f**). **Left**: temperature dependencies of *χ*_M_ (•) *χ*_M_*T* (ο); **right**: field dependences of magnetization per formula unit. The solid lines (red and blue) are calculated using the PHI program.

The molar magnetic susceptibility for **PPh_4_[*Nd*L_4_]**, **PPh_4_[*Dy*L_4_]** and **PPh_4_[*Tm*L_4_]** has been converted to the *χ_M_T* product, whose temperature dependence is displayed in Figure 6 on the left. The field dependence of the magnetization per formula unit *M*_1_ = *M*_mol_/*N*_A_*µ*_B_ at constant temperature is shown in Figure 6 on the right.

The room-temperature *χ*_M_*T* values are 17.27 cm^3^Kmol^−1^ for **PPh_4_[*Dy*L_4_]**, 7.20 cm^3^Kmol^−1^ for **PPh_4_[*Tm*L_4_]**, and 1.98 cm^3^Kmol^−1^ for **PPh_4_[*Nd*L_4_]**, which are a little bit higher than the expected values of 14.17 cm^3^Kmol^−1^ for a free *Dy^III^* ion (4f^9^, *J* = ^15^/_2_, *S* = ^5^/_2_, *L* = 5 with ^6^H_15/2_ ground term, g = ^4^/_3_), 7.18 cm^3^Kmol^−1^ for a free *Tm^III^* ion (4f^12^, *J* = 6, *S* = 1, *L* = 5 with ^3^H_6_ ground term, g = 1.17), and 1.65 cm^3^Kmol^−1^ for a free *Nd^III^* ion (4f^3^, *J* = ^9^/_2_, *S* = ^3^/_2_, *L* = 6 with ^4^I_9/2_ ground term, g = 0.73). These values steadily decrease and then rapidly descend below 50 K (25 K for **PPh_4_[*Tm*L_4_]**) owing to the thermal depopulation of excited Stark sublevels and/or antiferromagnetic intermolecular interactions transmitted between lanthanide ions in the crystal lattice. The fitting of the magnetic data (χ*T*(*T*)) of such complexes was carried out using the well–known PHI program [41], taking into account the possibility of intermolecular interaction and the terms of the spin–orbit constant λ of paramagnetic lanthanide ions (−357 *Dy^III^*, −684 *Tm^III^*, 299 *Nd^III^*). The calculated curve matches the magnetic data well (the solid lines in Figure 6). The very small z*J*’ values obtained (−0.014 cm^−1^ for **PPh_4_[*Dy*L_4_]**, −0.023 cm^−1^ for **PPh_4_[*Tm*L_4_]**, −0.30 cm^−1^ for **PPh_4_[*Nd*L_4_]**) clearly show that antiferromagnetic interactions between *Ln^III^* ions in the crystal lattice are considered to be relatively weak (practically negligible) due to the rather long *Ln*—*Ln* distance (the shortest is 13.51 Å) and efficient shielding effect of unpaired electrons in the *Ln*-4f orbitals, thereby making small contributions to the magnetization process.

The field-dependent magnetization data for these complexes exhibit continuous increases up to 7.15 μ*_B_* for **PPh_4_[*Dy*L_4_]**, 3.55 μ*_B_* for **PPh_4_[*Tm*L_4_]**, and 1.16 μ*_B_* for **PPh_4_[*Nd*L_4_]** (at 2 K) or 7.09 for **PPh_4_[*Dy*L_4_]**, 3.49 μ*_B_* for **PPh_4_[*Tm*L_4_]**, and 0.87 μ*_B_* for **PPh_4_[*Nd*L_4_]** (at 5 K, Figure 6) right per formula unit *M*_1_ = *M*_mol_/*N*_A_*μ*_B_. The lack of high-field saturation suggests the presence of significant magnetic anisotropy. The small differences between the values of magnetization for an increasing magnetic field and decreasing magnetic field at 2 K can be a result of torquing effects in the sample.

#### 2.4.2. AC Susceptibility

**Figure 7:**
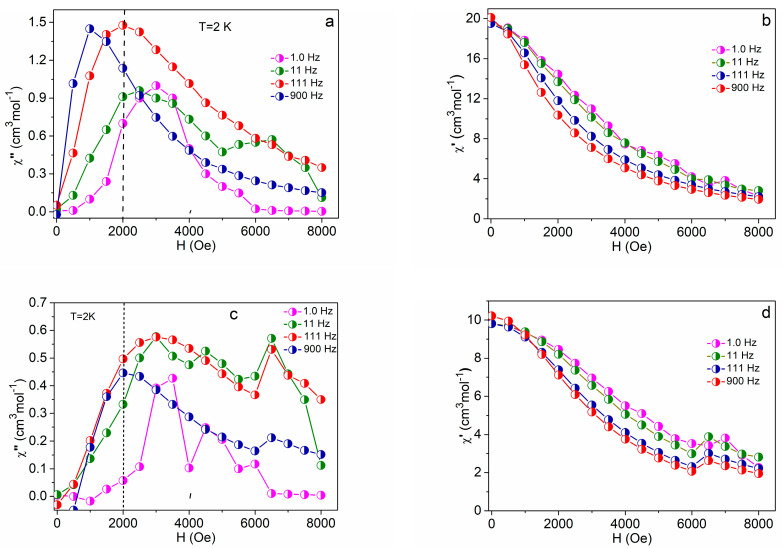

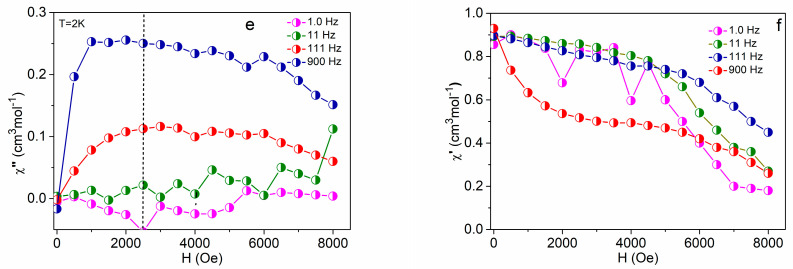
Field scan of AC susceptibility for (**a**) **PPh_4_[*Dy*L_4_]** (**a**,**b**), **PPh_4_[*Tm*L_4_]** (**c**,**d**), and **PPh_4_[*Nd*L_4_]** (**e**,**f**) at *T* = 2.0 K for four trial frequencies. Vertical marks indicate the frequencies selected for detailed mapping. Lines—a guide to the eye.

AC susceptibility data were acquired first at T = 2.0 K for a set of representative frequencies of the alternating field (f = 1.0, 11, 1111, and 1116 Hz) by ramping the magnetic field from zero to *B*_DC_ = 0.8 T or even higher; the working amplitude *B*_AC_ = 0.3 mT was used. For the examined compounds (*Dy*, *Tm*, *Nd*), there is no absorption signal (out-of-phase susceptibility component *χ*″) at the zero magnetic field owing to fast magnetic tunneling. The out-of-phase susceptibility *χ*″ increases with the applied field up to a maximum and then attenuates (Figure 7). The graph shows the regions in which individual frequencies yield the maximum response and indicates that **PPh_4_[*Dy*L_4_], PPh_4_[*Tm*L_4_]**, and **PPh_4_[*Nd*L_4_]** can exhibit field-induced slow magnetic relaxation.

**Figure 8:**
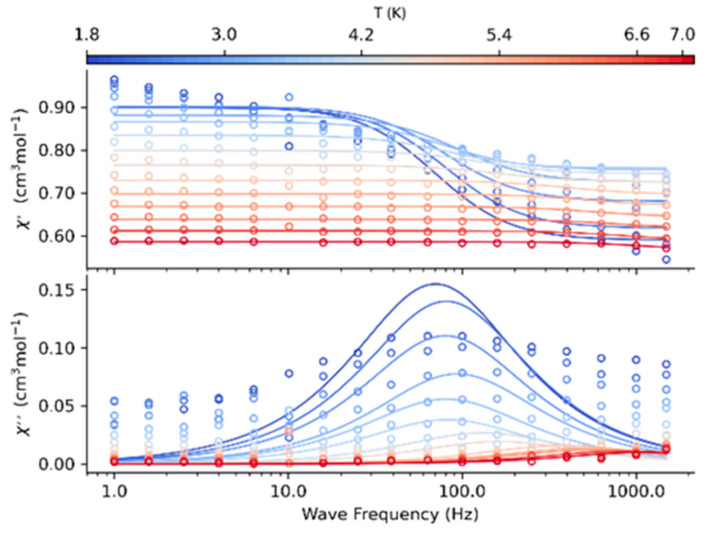
Frequency scan of AC susceptibility for **PPh_4_[*Dy*L_4_]** at fixed *B*_DC_ for varied *T*. Lines—fitted.

The next scans of mapping magnetic response were conducted at a fixed external magnetic field *B*_DC_ = 0.2 T (for **PPh_4_[*Dy*L_4_]** and **PPh_4_[*Tm*L_4_]**) and 0.25 T (for **PPh_4_[*Nd*L_4_]**), changing the frequency between *f* = 0.1 and 1500 Hz for a set of temperatures between *T* = 1.8 and 7 K. The curves for both **PPh_4_[*Tm*L_4_]** and **PPh_4_[*Nd*L_4_]** display the frequency dependence of magnetic susceptibilities in the out-of-phase mode, but do not show an onset of peaks (Figure S8). The frequency dependences of the AC susceptibility components at different temperatures for **PPh_4_[*Dy*L_4_]** shown in Figure 8 are evidence of one relaxation channel with a well-developed peak at 2 K and *B*_DC_ = 0.2 T with the maximum located at *f* = 96 Hz in the out-of-phase susceptibility curve. These data were fitted using CC-FIT2 software [42] by employing the simple Debye model (Table S5). Experimental curves without maxima were omitted. The quality of the fit is controlled by the standard deviation for each parameter and the discrepancy factors *R*(*χ*′) and *R*(*χ*″). Based upon the fitted parameters, the overall *χ*′ vs. *f* and *χ*″ vs. *f* curves were calculated, as displayed by solid lines in Figure 8. These pass through the experimental points.

The temperature evolution of the relaxation time has been fitted by a nonlinear relaxation equation due to reciprocating thermal behavior:

*τ^−^*^1^ = *τ_o_*^−1^ exp(*U*/*k_B_T*) + *CT^n^* + *AB*^m^*T* + *D*_t_

where the barrier to spin reversal *U* and the extrapolated relaxation time τ_0_ refer to the Orbach process; *A* characterizes the direct process; *C*—the Raman process; and *D_t_*—temperature-independent quantum tunneling. The resulting set of parameters *U* = 29(4) K, τ_0_ = 1.99·10^−6^ s, and t_QTM_ = 2.09·10^−2^ s shows that at higher temperatures, the Orbach process dominates and refers to climbing over the barrier to spin reversal, and at the lowest temperatures, the quantum tunneling relaxation process takes on significance (with the omission of the Raman and direct processes due to unrealistic n and m parameters; see inset in Figure S9). In terms of reciprocating thermal behavior, on further cooling, the high-frequency relaxation time is shortened instead of increased, and this is documented in at least a few examples of mononuclear *Mn^II^*, *Co^II^*, *Ni^II^*, and *Cu^II^* chelates and a lanthanide-containing complex of *Gd^III^* and *Dy^III^*.

## 3. Experimental Section

### 3.1. Methods

IR spectra of the obtained compounds were recorded on a Perkin Elmer Spectrum BX spectrometer using KBr pellets (Figure S1).

Emission spectra of the obtained coordination compounds were measured on a ≪Fluorolog FL 3-22≫ spectrofluorometer at 298 K. Excitation spectra at 293 and 77 K as well as emission spectra at 77 K were obtained using an Edinburgh Instruments FLSP 920 spectrofluorometer equipped with a 450 W continuous xenon arc lamp, single-grating excitation and emission monochromators, and a Single Photon Counting photomultiplier (R13456, Hamamatsu, Japan). The spectra were corrected for the instrument’s response. Emission decay times were measured with a μF920 H 60 W xenon lamp as the excitation source, triggered by the spectrometer controller (Edinburgh Instruments FLSP 920 spectrofluorometer). Measurements at 77 K were taken in a home-made quartz dewar cooled by liquid nitrogen. The emission lifetimes of ^4^F_3/2_ (**PPh_4_[*Nd*L_4_]**) and ^1^G_4_ (**PPh_4_[*Nd*L_4_]**) were too short for measurements using our equipment. Mechanoluminescence was recorded on an Ocean Optics spectrometer with a CCD camera using an optical fiber.

The DC magnetic data were collected using the SQUID magnetometer (MPMS, Quantum Design) calibrated with a palladium rod (Materials Research Corporation, purity 99.9985%) with ca 29.2 mg of sample. Magnetic measurements were carried out by crushing the crystals and restraining the sample in order to prevent any displacement. The susceptibility data were acquired at *B*_DC_ = 0.1 T between *T* = 1.8 and 300 K.

X-ray diffraction data for **PPh_4_[*Ln*L_4_]** (*Ln* = *Nd*, *Sm*, *Dy*) were collected on a Bruker APEX-II CCD diffractometer with graphite-monochromated MoKα radiation (λ = 0.71073 Å). The structures were solved with the SHElXT [43] solution program using Olex2 [44] as the graphical interface. The structures were refined with SHElXL using full-matrix least-squares minimization on F^2^. All non-hydrogen atoms were refined within anisotropic approximation. The positions of the hydrogen atoms were located based on electron density difference maps and refined by the “riding” model with *U*_iso_ = 1.2*U*_eq_ of the carrier atom. In the structure **PPh_4_[*Nd*L_4_]**, the phenyl rings at atoms C1, C39, C89, and P7 are disordered over two positions, A and B, with equal populations. **PPh_4_[*Tm*L_4_]** is isostructural with **PPh_4_[*Nd*L_4_].**

Atomic coordinates, bond lengths, and angles as well as displacement parameters have been deposited into the Cambridge Crystallographic Data Centre, 11 Union Road, Cambridge, CB2 1EZ, UK (E-mail: deposit@ccdc.cam.ac.uk; fax: +44 1223 336033). These data can also be obtained free of charge via https://www.ccdc.cam.ac.uk/structures/ (accessed on 6 January 2025). Deposition numbers CCDC 2392149 (for **PPh_4_[*Nd*L_4_]**), 23192150 (for **PPh_4_[*Sm*L_4_]**), and 23192151 (for **PPh_4_[*Dy*L_4_]**) contain the supplementary crystallographic data for this paper. The crystallographic data and experimental parameters for the complexes are given in Table S1.

X-ray diffraction spectra of polycrystalline samples were collected on a Shimadzu XRD-6000 diffractometer (Shimadzu, Kyoto, Japan) with a graphite monochromator in front of the counter with a scan step 0.05° in 2*θ* range 5–50°.

### 3.2. Synthesis

Syntheses of diphenyl-N-benzoylamidophosphate, its sodium salt NaL, and the complexes **PPh_4_[*Ln*L_4_]** were performed using the methods described in [20]. The complexes were obtained according to the following scheme:

Ln(NO_3_)_3_·nH_2_O + 4 NaL + PPh_4_Br → PPh_4_[*Ln*L_4_] + 3 NaNO_3_↓ + NaBr↓ + n H_2_O

(*Ln*^3+^ = *Nd*, *Sm*, *Gd*, *Dy*, *Tm*)

The single crystals for X-ray analysis were obtained from the complexes’ solutions in a mixture of the solvents acetone and 2-propanol by slow evaporation of the solvents at room temperature.

Compound **PPh_4_[*Nd*L_4_]**: Elemental anal. Calcd (%): H 4.46%, C 63.32%, N 2.95%; found H 4.44%, C 62.64%, N 3.07%. IR (KBr, cm^−1^): 3062 w, 2922 w, 1592 m, 1526 s (ν(C=O)), 1488 s, 1441 w, 1388 s, 1199 s (ν(P=O)), 1150 m, 1106 m, 1069 w, 1026 w, 1004 w, 958 s, 930 s, 900 m, 829 m, 771 m, 758 m, 715 m, 688 m, 594 w, 526 s.

Compound **PPh_4_[*Sm*L_4_]**: Elemental anal. Calcd (%): H 4.45%, C 63.12%, N 2.94%; found H 4.46%, C 62.23%, N 2.97%. IR (KBr, cm^−1^): 3064 w, 2926 w, 1591 m, 1525 s (ν(C=O)), 1483 s, 1437 w, 1390 s, 1202 s (ν(P=O)), 1154 m, 1109 m, 1066 w, 1023 w, 1003 w, 958 s, 928 s, 900 m, 831 m, 775 m, 754 m, 716 m, 689 m, 596 w, 529 s.

Compound **PPh_4_[*Gd*L_4_]**: Elemental anal. Calcd (%): H 4.43%, C 62.89%, N 2.94%; found H 4.52%, C 61.98%, N 3.02%. IR (KBr, cm^−1^): 3063 w, 2925 w, 1591 m, 1526 s (ν(C=O)), 1483 s, 1439 w, 1390 s, 1201 s (ν(P=O)), 1154 m, 1109 m, 1067 w, 1025 w, 1003 w, 958 s, 930 s, 901 m, 832 m, 775 m, 755 m, 716 m, 689 m, 596 w, 528 s.

Compound **PPh_4_[*Dy*L_4_]**: Elemental anal. Calcd (%): H 4.42%, C 62.71%, N 2.93%; found H 4.60%, C 61.98%, N 2.93%. IR (KBr, cm^−1^): 3062 w, 2924 w, 1591 m, 1530 s (ν(C=O)), 1489 s, 1443 w, 1389 s, 1202 s (ν(P=O)), 1155 m, 1108 m, 1068 w, 1026 w, 1004 w, 960 s, 931 s, 902 m, 835 m, 773 m, 757 m, 714 m, 689 m, 597 w, 527 s.

Compound **PPh_4_[*Tm*L_4_]**: Elemental anal. Calcd (%): H 4.41%, C 62.51%, N 2.92%; found H 4.47%, C 61.83%, N 3.01%. IR (KBr, cm^−1^): 3064 w, 2924 w, 1591 m, 1535 s (ν(C=O)), 1489 s, 1442 w, 1390 s, 1202 s (ν(P=O)), 1154 m, 1105 m, 1066 w, 1023 w, 1005 w, 960 s, 931 s, 900 m, 833 m, 773 m, 754 m, 712 m, 689 m, 597 w, 526 s.

The PXRD patterns of the complexes (Figure S2) are in good agreement with the calculated patterns of the single crystal. The calculated PXRD patterns for the single crystals of **PPh_4_[*Nd*L_4_]** and **PPh_4_[*Dy*L_4_]** are almost identical. The main observed difference between the calculated patterns is a slight shift in the majority of peaks of the dysprosium complex’s single crystal toward higher values of 2*θ* for 0.1–0.2. Such a small difference does not allow an objective assessment of the purity of the phases. The positions of peaks in the experimental PXRD patterns of the studied complexes differ for 0.05–0.1 2*θ.* The presence of both types of crystal in each of the samples cannot be excluded.

## 4. Conclusions

In the **PPh_4_[*Ln*L_4_]** (*Ln* = *Nd^III^*, *Sm^III^*, *Dy^III^*, *Tm^III^*)-type coordination compounds, which crystallize in the tetragonal space group I$\bar{4}$ (*Nd*, *Tm*) and the monoclinic space group *I*2 (*Sm*, *Dy*), the bidentate ligand completely fills the first coordination sphere of the *Ln^III^* ions. This, combined with the appropriate energy of the ligand triplet state, results in the studied chelates having intense sensitized emission, and the well-designed ligand is an effective sensitizer of this luminescence. The emission lifetimes in the visible range for **PPh_4_[*Sm*L_4_]** and **PPh_4_[*Dy*L_4_]** have large values and are mono-exponential in nature. At the same time, due to the elimination of high-energy vibrations from the six-membered chelate rings, the process of multiphonon emission quenching was reduced, which resulted in the presence of emission not only in the visible range but also in the NIR region. Thus, the compounds *Sm*, *Dy*, and *Tm* are characterized by dual Vis and NIR emission from the range of the first, second, and third telecommunication windows as well as the first and second biological windows, which makes them interesting from an application point of view. An unusual behavior of the **PPh_4_[*Tm*L_4_]** compound is also shown, where the nature of the emission excitation spectrum is dependent on the emission wavelength and temperature. **PPh_4_[*Dy*L_4_]**, **PPh_4_[*Sm*L_4_]**, and **PPh_4_[*Tm*L_4_]** show intense mechanoluminescence visible to the naked eye. To our knowledge, this is the first report of mechanoluminescence for the Tm^III^ coordination compound. **PPh_4_[*Nd*L_4_]** and **PPh_4_[*Tm*L_4_]** exhibit field-induced slow magnetic relaxation. In addition, the **PPh_4_[*Dy*L_4_]** compound has multifunctional properties, exhibiting mechanoluminescence and SIM behavior (*U* = 29(4) K, τ_0_ = 1.99·10^−6^ s, t_QTM_ = 2.09·10^−2^ s). Due to its bright mechanoluminescence, it is potentially interesting as an impact sensor and as a model compound to study the dependence of quantitative mechanoluminescence parameters and SIM properties on the structural properties of the coordination compound, also with a change in the counterion.

## Figures and Tables

**Table 1:** Selected geometrical parameters for **[*Ln*L_4_]**^−^ (*Ln* = *Nd*, *Sm*, *Dy*) complex anions.

Parameters	PPh_4_[*Nd*L_4_]		PPh_4_[*Sm*L_4_]			PPh_4_[*Dy*L_4_]		
	Anion 1	Anion 2	Anion 1	Anion 2	Anion 3	Anion 1	Anion 2	Anion 3
d(*Ln*–O(P)), Å	2.426(6) 2.427(6) 2.471(6) 2.455(6)	2.449(9)	2.423(11) 2.391(9) 2.449(8) 2.367(10)	2.421(10) 2.392(9) 2.394(9) 2.428(11)	2.440(9) 2.373(8)	2.375(11) 2.355(10) 2.444(10) 2.350(10)	2.345(11) 2.256(10) 2.357(10) 2.366(10)	2.378(10) 2.303(10)
d(*Ln*–O(C)), Å	2.463(6) 2.444(7) 2.425(6) 2.466(7)	2.438(11)	2.445(8) 2.420(9) 2.377(10) 2.408(9)	2.428(10) 2.489(9) 2.433(11) 2.417(9)	2.425(8) 2.447(8)	2.440(9) 2.366(12) 2.356(11) 2.346(10)	2.380(10) 2.454(10) 2.331(10) 2.425(9)	2.394(10) 2.359(9)
*Ln^III^* coordination polyhedron shape (the closest) ^1^	Triangular dodecahedron	Triangular dodecahedron	Square antiprism	Triangular dodecahedron	Square antiprism	Square antiprism	Triangular dodecahedron	Square antiprism

^1^ According to the smallest value of the convergence factors.

## Data Availability

The original contributions presented in the study are included in the article/Supplementary Materials; further inquiries can be directed to the corresponding authors.

## Supplementary Materials

The following supporting information can be downloaded at https://www.mdpi.com/article/10.3390/molecules30061245/s1, Table S1. Crystal data and structure refinement for **PPh_4_[*Ln*L_4_]** (*Ln* = *Nd*, *Sm*, *Dy*); Table S2. Criteria of coordination polyhedron determination for coordination number of 8; Table S3. The conformational characteristics of metal cycles in **[*Ln*L_4_]**^−^ (*Ln* = *Nd*, *Sm*, *Dy*) anions; Table S4. Intra- and intermolecular interactions in the complexes; Table S5. Results of the fitting procedure for AC susceptibility components of 1 at BDC = 0.2 with a Debye model; Figure S1. IR spectra of the obtained complexes: 1—**PPh_4_[*Nd*L_4_]**; 2—**PPh_4_[*Sm*L_4_]**; 3—**PPh_4_[*Dy*L_4_]**; 4—**PPh_4_[*Tm*L_4_]**; Figure S2. PXRD patterns of the obtained complexes: 1—**PPh_4_[*Nd*L_4_]**; 2—**PPh_4_[*S*mL_4_]**; 3—**PPh_4_[*Dy*L_4_]**; 4—**PPh_4_[*Tm*L_4_]**; Figure S3. A schematic diagram of the energy levels of the studied lanthanides vs. LLTS. Figure S4. Luminescence excitation spectra of **PPh_4_[*Tm*L_4_**] at 300 and 77 K, λ_em_ = 472.5 nm (300 K), λ_em_ = 480.2 nm (77 K); Figure S5. Reflectance spectra of **PPh_4_[*Ln*L_4_]** compounds in the solid state undiluted and diluted with BaSO_4_ at 300 K. Figure S6. Luminescence excitation spectra of **PPh_4_[*Nd*L_4_]** at 300 K obtained for different holders, λ_em_ = 1055 nm; Figure S7. CIE 1931 xy chromaticity diagram for **PPh_4_[*Sm*L_4_]**, **PPh_4_[*Dy*L_4_]**, and **PPh_4_[*Tm*L_4_]** compounds; Figure S8. The AC susceptibility data for compounds (a) **PPh_4_[*Tm*L_4_]** and (b) **PPh_4_[*Nd*L_4_]** at the external DC field; Figure S9. Arrhenius-like plot for **PPh_4_[*Dy*L_4_]**. Video showing intense yellow mechanoluminescence of **PPh_4_[*Dy*L_4_]**.
