# Supplementary material for: Dual Visible and NIR Emission, Mechanoluminescence, and Magnetic Properties of PPh4[LnL4] Chelates with Diphenyl-N-Benzoylamidophosphate
Source: Molecules. 2025 Mar 10;30(6):1245. doi: 10.3390/molecules30061245 (PMC11946015; doi:10.3390/molecules30061245)
Supplement: Supplementary file 1 [file molecules-30-01245-s001.zip › molecules-3441632-supplementary.pdf]

Supporting information

N.S. Kariaka[a,*], D. K. Panasiuk[a], V.A. Trush[a], S.S. Smola[b], N.V. Rusakova[b], V.V. Dyakonenko[c], S.V. Shishkina[c,e], A. Lipa[d], A. Bienko[d], J. Nasalska[d], P. Gawryszewska[d,*], V.M. Amirkhanov[a]

[a] *Taras Shevchenko National University of Kyiv, Department of Chemistry, Volodymyrska str. 64, 01601 Kyiv, Ukraine*

[b] *A.V. Bogatsky Physicochemical Institute, National Academy of Sciences of Ukraine, 86 Lustdorfska doroga, 65080 Odesa, Ukraine*

[c] *SSI "Institute for Single Crystals", National Academy of Sciences of Ukraine Nauky ave. 60, 61001 Kharkiv, Ukraine*

[d] *Faculty of Chemistry, University of Wroclaw, 14 F. Joliot-Curie Str., 50-383, Wroclaw, Poland*

[e] *Institute of Organic Chemistry, National Academy of Sciences of Ukraine, 5, Academician Str., Kyiv 02660, Ukraine*

Contents

Table S1. Crystal data and structure refinement for **$PPh_4[LnL_4]$** (Ln = Nd, Sm, Dy). 3

Table S2. Criteria of coordination polyhedron determination for 8 coordination number. 4

Table S3. The conformational characteristics of metal cycles in **$[LnL_4]$- (Ln=Nd, Sm, Dy)** anions. 5

Table S4. Intra- and intermolecular interactions in the complexes. 6

Table S5. Results of the fitting procedure for AC susceptibility components of **$PPh_4[DyL_4]$** at BDC = 0.2 with a Debye model...9

Figure S1. IR spectra of the obtained complexes: **1 – $PPh_4[NdL_4]$, 2 – $PPh_4[SmL_4]$, 3 - $PPh_4[GdL_4]$, 4 - $PPh_4[DyL_4]$, 5 - $PPh_4[TmL_4]$** 10

Figure S2. PXRD patterns of the obtained complexes**: 1 – $PPh_4[NdL_4]$, 2 – $PPh_4[SmL_4]$, 3 - $PPh_4[GdL_4]$, 4 - $PPh_4[DyL_4]$, 5 - $PPh_4[TmL_4]$**...10

Figure S3. A schematic diagram of the energy levels of the studied lanthanides vs LLTS. 11

Figure S4. Luminescence excitation spectra of **$PPh_4[TmL_4]$** at 300 and 77 K, λ_{em} = 472.5 nm (300 K), λ_{em} = 480.2 nm (77 K)**.** The band marked with an asterisk appears to be an artifact, in this spectral range the Tm^{III} ion has no absorption band...11

Figure S5. Reflectance spectra of **$PPh_4[LnL_4]$** compounds in the solid state undiluted and diluted with $BaSO_4$ at 300 K. 12

Figure S6. Luminescence excitation spectra of **$PPh_4[NdL_4]$ at 300 K** obtained for different holders, λ_{em} = 1055 nm...12

Figure S7. CIE 1931 xy chromaticity diagram for **$PPh_4[SmL_4]$, $PPh_4[DyL_4]$** and **$PPh_4[TmL_4]$** complexes. 13

Figure S8. The AC susceptibility data for compounds: a) **$PPh_4[TmL_4]$** and b) **$PPh_4[NdL_4]$** at external DC field: left - temperature dependence of the out-of-phase molar susceptibility; right - temperature dependence of the in-phase molar susceptibility...14

Figure S9. Arrhenius–like plot for **$PPh_4[DyL_4]$.**...15

Table S1. Crystal data and structure refinement for **PPh4[LnL4]** (Ln = Nd, Sm, Dy).

	**PPh4[NdL4]**	**PPh4[SmL4]**	**PPh4[DyL4]**
Chemical formula	$C_{100}H_{80}N_4O_{16}P_5Nd$	$C_{100}H_{80}N_4O_{16}P_5Sm$	$C_{100}H_{80}N_4O_{16}P_5Dy$
Mr	1892.77	1898.88	1911.03
Crystal system, space group	Tetragonal, $I\bar{4}$	Monoclinic, *I*2	Monoclinic, *I*2
Temperature (K)	173.15	173.15	173.15
a, b, c (Å)	39.3565(18), 39.3565(18), 14.6197(8)	38.8985(16), 14.5282(6), 39.0976(17)	38.958(3), 14.4621(9), 39.073(3)
α, β, γ (°)	90, 90, 90	90, 90.078(5), 90	90, 90.056(9), 90
V (Å^3)	22645(2)	22095.0(16)	22014(3)
Z	10	10	10
μ (mm^{-1})	0.731	0.826	1.011
D, r/cm^3	1.388	1.427	1.441
F(000)	9710	9730	9770
T_{min}, T_{max}	0.759, 0.931	0.6225, 0.7461	0.688, 0.906
No. of measured, independent, observed [I > 2□(I)] reflections	165880, 26014, 15440	353108, 49344, 44225	346230, 50510, 44561
Goodness of fit on F^2	1.045	1.048	0.998
Final R indexes [all data]	R_1 = 0.1433, wR_2 = 0.1693	R_1 = 0.0747, wR_2 = 0.1485	R_1 = 0.0806, wR_2 = 0.1620
Final R indices [I > 2□(I)]	R_1=0.0762, wR_2=0.1452	R_1=0.0655, wR_2=0.1427	R_1=0.0714, wR_2=0.1550
Flack parameter	-0.010(5)	0.079(4)	0.133(5)
CCDC reference number	2392149	23192150	23192151

Table S2. Criteria of coordination polyhedron determination for 8 coordination number.

Parameters	$PPh_4[NdL_4]$		$PPh_4[SmL_4]$			$PPh_4[DyL_4]$		
	Anion 1	Anion 2	Anion 1	Anion 2	Anion 3	Anion 1	Anion 2	Anion 3
Octagon (D_{8h})	30.538	32.803	29.624	31.073	29.878	29.610	30.935	30.216
Heptagonal pyramid (C_{7v})	23.608	24.648	23.419	23.777	23.764	23.481	24.076	23.971
Hexagonal bipyramid (D_{6h})	15.552	17.009	14.645	16.014	15.106	14.882	16.236	14.858
Cube (O_h)	10.679	9.779	10.070	10.586	10.377	9.739	11.008	10.115
Square antiprism (D_{4d})	1.322	3.055	**0.855**	1.627	**0.807**	**0.838**	1.664	**0.968**
Triangular dodecahedron (D2d)	**0.884**	**0.304**	1.618	**0.587**	1.369	1.684	**0.677**	1.333
Johnson gyrobifastigium J26 (D2d)	12.537	12.433	12.817	12.710	12.936	13.355	12.776	12.690
Johnson elongated triangular bipyramid J14 (D3h)	28.194	30.174	27.941	29.312	28.606	28.292	28.865	29.068
Biaugmented trigonal prism J50 (C2v)	2.220	3.444	2.046	2.504	2.371	1.970	2.404	2.350
Biaugmented trigonal prism (C2v)	1.627	2.771	1.536	1.926	1.836	1.458	1.909	1.838
Snub diphenoid J84 (D2d)	3.271	2.899	3.828	2.772	3.499	3.874	2.552	3.307
Triakis tetrahedron (Td)	11.525	10.595	10.752	11.325	11.227	10.397	11.609	10.967
Elongated trigonal bipyramid (D3h)	23.080	24.188	23.261	24.323	23.513	24.009	24.851	24.032

Table S3. The conformational characteristics of metal cycles in **[LnL4]- (Ln=Nd, Sm, Dy)** anions.

Mean plane/atoms	Mean plane accuracy, Å	Atom/deviation from plane, Å
**$[Nd(L)_4]^-$**		
N1…P1…O1…Nd1	0.01	C1 -0.22<br>O2 -0.44
N2…P2…O5…Nd1	0.02	C20 0.27<br>O6 0.48
N3…P3…O9…Nd1	0.03	C39 0.29<br>O10 0.72
O14…Nd1…O13…P4	0.02	C58 0.45<br>N4 0.60
O18…Nd2…O17…P5	0.02	C89 -0.40<br>N5 -0.49
**$[Sm(L)_4]^-$**		
N1…P1…O2…Sm1	0.01	C1 0.15<br>O1 0.27
O5…Sm1…O6…P2	0.01	C20 0.45<br>N2 0.54
P3…O10…Sm1…O9	0.02	C39 -0.61<br>N3 -0.73
P4…O14…Sm1…O13	0.02	C58 0.27<br>N4 0.22
O17…C77…N5…P5	0.02	O18 -0.53<br>Sm2 -1.17
P6…O22…Sm2…O21	0.02	N6 -0.47<br>C96 -0.23
P7…O25…Sm2…O25	0.01	N7 0.48<br>C115 0.41
P8…O30…Sm2…O29	0.02	N8 0.70<br>C134 0.51
P9…O34…Sm3…O33	0.02	N9 -0.64<br>C153 -0.53
O37…C172…N10…P10	0.02	O38 0.19<br>Sm3 0.59
**$[Dy(L)_4]^-$**		
P1…O2…Dy1…O1	0.02	N1 0.27<br>C1 0.20
O5…Dy1…P2…O6	0.01	N2 -0.50<br>C20 -0.43
O9…Dy1…O10…P3	0.02	N3 0.71<br>C39 0.61
O13…Dy1…O14…P4	0.02	N4 -0.21<br>C58 -0.32
P5…N5…C77…O17	0.01	O18 0.48<br>Dy2 1.05
N6…C96…O21…Dy2	0.02	O22 -0.38<br>P6 -0.49
O25…Dy2…O26…P7	0.01	N7 -0.42<br>C115 -0.36
O29…Dy2…O30…P8	0.02	N8 -0.64<br>C134 -0.52
O33…Dy3…O34…P9	0.02	N9 0.58<br>C153 0.49
P10…N10…C172…O37	0.02	O38 -0.20<br>Dy3 -0.38

Table S4. Intra- and intermolecular interactions in the complexes.

Atom 1	Atom 2	Symmetry atom 1	Symmetry atom 2	Contact distance, Å
		**$PPh_4[NdL_4]$**		
H7B	H74	x,y,z	y,1-x,2-z	2.393
C51	H92A	x,y,z	1.5-y,1/2+x,1.5-z	2.703
H9	C86	x,y,z	x,y,z	2.866
H25	C82	x,y,z	x,y,z	2.788
H25	H82	x,y,z	x,y,z	2.289
O4	H98	x,y,z	x,y,z	2.565
C71	H100	x,y,z	x,y,z	2.845
C72	H100	x,y,z	x,y,z	2.811
C14	H106	x,y,z	x,y,-1+z	2.835
C17	H107	x,y,z	x,y,-1+z	2.882
C19	H106	x,y,z	x,y,-1+z	2.771
O5	H122	x,y,z	1-y,x,2-z	2.555
C58	H121	x,y,z	1-y,x,2-z	2.765
C59	H121	x,y,z	1-y,x,2-z	2.815
C20	H112	x,y,z	-1/2+y,1.5-x,2.5-z	2.881
C45	C42	x,y,z	x,y,z	2.748
C77	H132	x,y,z	x,y,z	2.668
C78	H132	x,y,z	x,y,z	2.852
C80	H131	x,y,z	x,y,z	2.783
C81	H131	x,y,z	x,y,z	2.895
C82	H132	x,y,z	x,y,z	2.850
O19	H128	x,y,z	-1/2+y,1.5-x,2.5-z	2.667
		**$PPh_4[SmL_4]$**		
C4	H25	x,y,z	x,-1+y,z	2.855
C12	H43	x,y,z	x,-1+y,z	2.833
C61	H44	x,y,z	x,-1+y,z	2.837
H9	C55	x,y,z	1.5-x,-1/2+y,1/2-z	2.794
H61	C47	x,y,z	1.5-x,-1/2+y,1/2-z	2.871
H61	H47	x,y,z	1.5-x,-1/2+y,1/2-z	2.348
H68	O28	x,y,z	x,-1+y,z	2.575
H68	C128	x,y,z	x,-1+y,z	2.882
C72	H100	x,y,z	x,-1+y,z	2.882
H23	C92	x,y,z	x,y,z	2.873
H5	C123	x,y,z	1-x,-1+y,1-z	2.844
C37	H130	x,y,z	1-x,-1+y,1-z	2.878
C65	H131	x,y,z	1-x,-1+y,1-z	2.791
C70	H131	x,y,z	1-x,-1+y,1-z	2.799
C30	H146	x,y,z	1-x,y,1-z	2.852
C12	H190	x,y,z	1.5-x,-1.5+y,1/2-z	2.814
C11	H190	x,y,z	1.5-x,-1.5+y,1/2-z	2.887
C32	H167	x,y,z	1.5-x,-1/2+y,1/2-z	2.845
H42	C167	x,y,z	1.5-x,-1/2+y,1/2-z	2.752
H42	C168	x,y,z	1.5-x,-1/2+y,1/2-z	2.869
O4	H193	x,y,z	1/2+x,-1/2+y,1/2+z	2.646
O4	H194	x,y,z	1/2+x,-1/2+y,1/2+z	2.719
C34	H195	x,y,z	1/2+x,-1/2+y,1/2+z	2.882
H30	C201	x,y,z	1/2+x,1/2+y,1/2+z	2.821
O16	H223	x,y,z	x,-1+y,z	2.646
O11	H230	x,y,z	1.5-x,-1/2+y,1/2-z	2.630
C14	H231	x,y,z	1.5-x,-1/2+y,1/2-z	2.825
C17	H232	x,y,z	1.5-x,-1/2+y,1/2-z	2.847
C67	H241	x,y,z	x,-1+y,z	2.888

C144	H114	x,y,z	1/2-x,-1/2+y,1/2-z	2.880
C148	H118	x,y,z	1/2-x,-1/2+y,1/2-z	2.871
C88	H184	x,y,z	x,-1+y,z	2.887
H89	C187	x,y,z	x,-1+y,z	2.894
H138	C183	x,y,z	x,-1+y,z	2.851
C105	H156	x,y,z	x,y,z	2.798
C104	H156	x,y,z	x,y,z	2.879
O32	C205	x,y,z	x,y,z	3.165
O32	H205	x,y,z	x,y,z	2.474
C103	H207	x,y,z	x,y,z	2.828
C104	H207	x,y,z	x,y,z	2.774
C147	H205	x,y,z	x,y,z	2.884
O27	H199	x,y,z	1/2-x,1/2+y,1/2-z	2.621
O27	H200	x,y,z	1/2-x,1/2+y,1/2-z	2.621
C147	H201	x,y,z	1/2-x,1/2+y,1/2-z	2.750
C148	H201	x,y,z	1/2-x,1/2+y,1/2-z	2.846
H87	C226	x,y,z	x,-1+y,z	2.858
H87	C225	x,y,z	x,-1+y,z	2.864
H93	H222	x,y,z	x,y,z	2.334
O17	H243	x,y,z	x,y,z	2.566
C130	H247	x,y,z	x,1+y,z	2.865
H130	H247	x,y,z	x,1+y,z	2.341
O39	H211	x,y,z	x,1+y,z	2.522
C180	H212	x,y,z	x,1+y,z	2.883
		**$PPh_4[DyL_4]$**		
H25	C4	x,y,z	x,-1+y,z	2.824
H43	C12	x,y,z	x,-1+y,z	2.848
H44	C61	x,y,z	x,-1+y,z	2.847
C47	H61	x,y,z	1/2-x,-1/2+y,1.5-z	2.858
H47	H61	x,y,z	1/2-x,-1/2+y,1.5-z	2.323
C55	C9	x,y,z	1/2-x,-1/2+y,1.5-z	3.400
C55	H9	x,y,z	1/2-x,-1/2+y,1.5-z	2.761
H23	C91	x,y,z	x,y,z	2.894
H23	C92	x,y,z	x,y,z	2.766
C68	H99	x,y,z	x,1+y,z	2.855
H68	O28	x,y,z	x,1+y,z	2.625
C72	H100	x,y,z	x,1+y,z	2.853
C30	H146	x,y,z	1-x,y,1-z	2.827
H34	H126	x,y,z	1-x,y,1-z	2.386
H5	C123	x,y,z	1-x,1+y,1-z	2.831
C65	H131	x,y,z	1-x,1+y,1-z	2.826
C70	H131	x,y,z	1-x,1+y,1-z	2.765
H32	C162	x,y,z	1/2-x,-1/2+y,1.5-z	2.859
H42	C167	x,y,z	1/2-x,-1/2+y,1.5-z	2.716
H42	C168	x,y,z	1/2-x,-1/2+y,1.5-z	2.841
C12	H190	x,y,z	1/2-x,1/2+y,1.5-z	2.851
C11	H190	x,y,z	1/2-x,1/2+y,1.5-z	2.866
H30	C201	x,y,z	x,-1+y,z	2.841
O4	H193	x,y,z	x,y,z	2.640
O4	H194	x,y,z	x,y,z	2.647
C34	H195	x,y,z	x,y,z	2.882
O16	H223	x,y,z	x,y,z	2.655
O11	C230	x,y,z	1/2-x,-1/2+y,1.5-z	3.219
O11	H230	x,y,z	1/2-x,-1/2+y,1.5-z	2.624
C14	H231	x,y,z	1/2-x,-1/2+y,1.5-z	2.828
C17	H232	x,y,z	1/2-x,-1/2+y,1.5-z	2.829

C113	H148	x,y,z	1.5-x,-1/2+y,1.5-z	2.897
C105	H156	x,y,z	x,-1+y,z	2.800
H105	H156	x,y,z	x,-1+y,z	2.397
H106	H178	x,y,z	x,y,z	2.387
H138	C183	x,y,z	x,y,z	2.891
O27	H199	x,y,z	1-x,-1+y,1-z	2.646
O27	H200	x,y,z	1-x,-1+y,1-z	2.659
C147	H201	x,y,z	1-x,-1+y,1-z	2.720
C148	H201	x,y,z	1-x,-1+y,1-z	2.825
O32	C205	x,y,z	1/2+x,-1/2+y,1/2+z	3.181
O32	H205	x,y,z	1/2+x,-1/2+y,1/2+z	2.520
C103	H207	x,y,z	1/2+x,-1/2+y,1/2+z	2.817
C104	H207	x,y,z	1/2+x,-1/2+y,1/2+z	2.750
C147	H205	x,y,z	1/2+x,-1/2+y,1/2+z	2.852
H93	H222	x,y,z	x,-1+y,z	2.313
H87	C226	x,y,z	x,y,z	2.890
C130	H247	x,y,z	x,-1+y,z	2.820
H130	H247	x,y,z	x,-1+y,z	2.287
O17	H243	x,y,z	x,y,z	2.596
O26	H243	x,y,z	x,y,z	2.699
O39	H211	x,y,z	1/2+x,-1/2+y,1/2+z	2.575
C180	H212	x,y,z	1/2+x,-1/2+y,1/2+z	2.831
C166	H213	x,y,z	1/2-x,-1/2+y,1.5-z	2.840
C167	H213	x,y,z	1/2-x,-1/2+y,1.5-z	2.814
C169	C214	x,y,z	1/2-x,-1/2+y,1.5-z	3.377
C180	H237	x,y,z	x,y,z	2.834

Table S5. Results of the fitting procedure for AC susceptibility components of **$PPh_4[DyL_4]$** at BDC = 0.2 with a Debye model.

T/K	*τ / s*	*R [τ]*	*χS*	*R [χS]*	*χT*	*R [χT]*
1.8	0.00226481	0.00033449	0.58984454	0.01666545	0.89989469	0.01407151
2.2	0.00196759	0.00027448	0.61772153	0.01456386	0.89801095	0.01178539
2.6	0.00198932	0.00032210	0.68016276	0.01330152	0.90112880	0.01079992
3.0	0.00170131	0.00031615	0.72533695	0.01101513	0.88056374	0.00852375
3.4	0.00196477	0.00040474	0.75350660	0.00858450	0.86536153	0.00694373
3.8	0.00192846	0.00043934	0.75771179	0.00649907	0.83404230	0.00522706
4.2	0.00120080	0.00029322	0.74457014	0.00529871	0.79826250	0.00386186
4.6	0.00098112	0.00027331	0.72570495	0.00457184	0.76444208	0.00295342
5.0	0.00041021	0.00009910	0.69662824	0.00406391	0.72894925	0.00183823
5.4	0.00031187	0.00007059	0.66994656	0.00347616	0.69732274	0.00141510
5.8	0.00028055	0.00009335	0.64372847	0.00470784	0.66808091	0.00172891
6.2	0.00020239	0.00005679	0.61726966	0.00372957	0.63789300	0.00109386
6.6	0.00018514	0.00002912	0.58883627	0.00235633	0.61146631	0.00066787
7.0	0.00013789	0.00002543	0.56559917	0.00274287	0.58600253	0.00059196

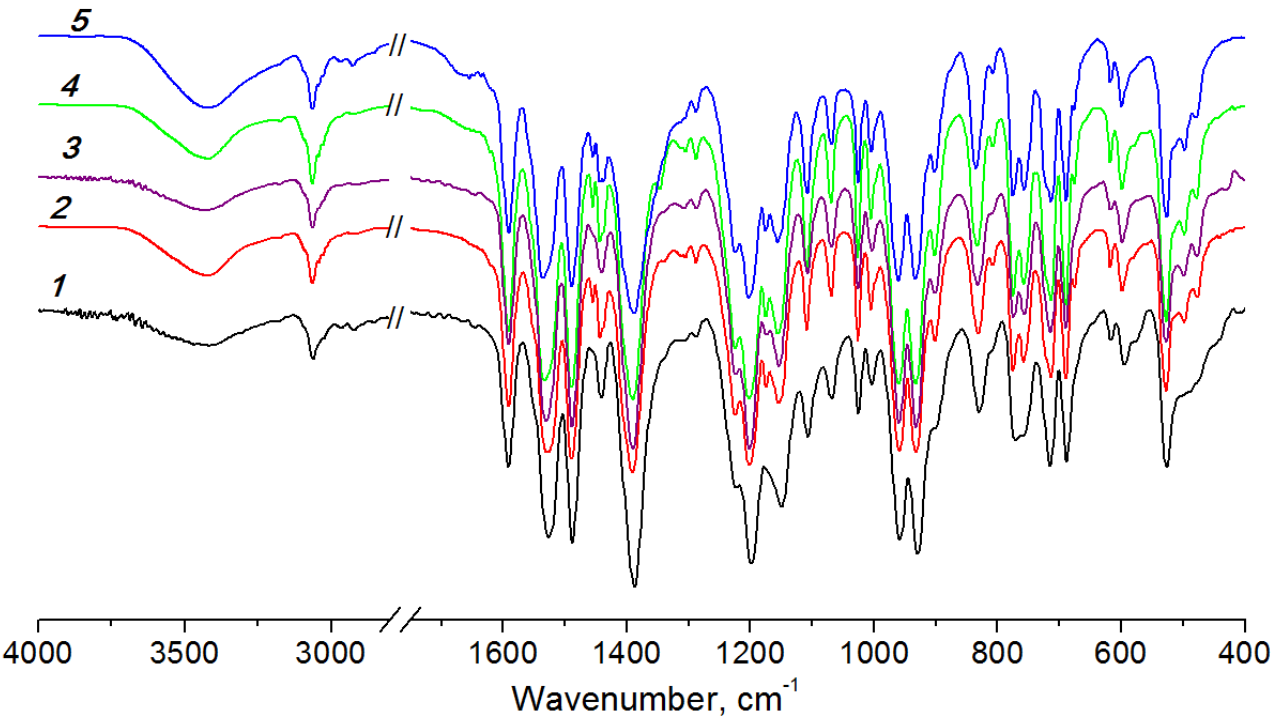

Figure S1. IR spectra of the obtained complexes: **1 – $PPh_4[NdL_4]$, 2 – $PPh_4[SmL_4]$, 3 - $PPh_4[GdL_4]$, 4 - $PPh_4[DyL_4]$, 5 - $PPh_4[TmL_4]$**

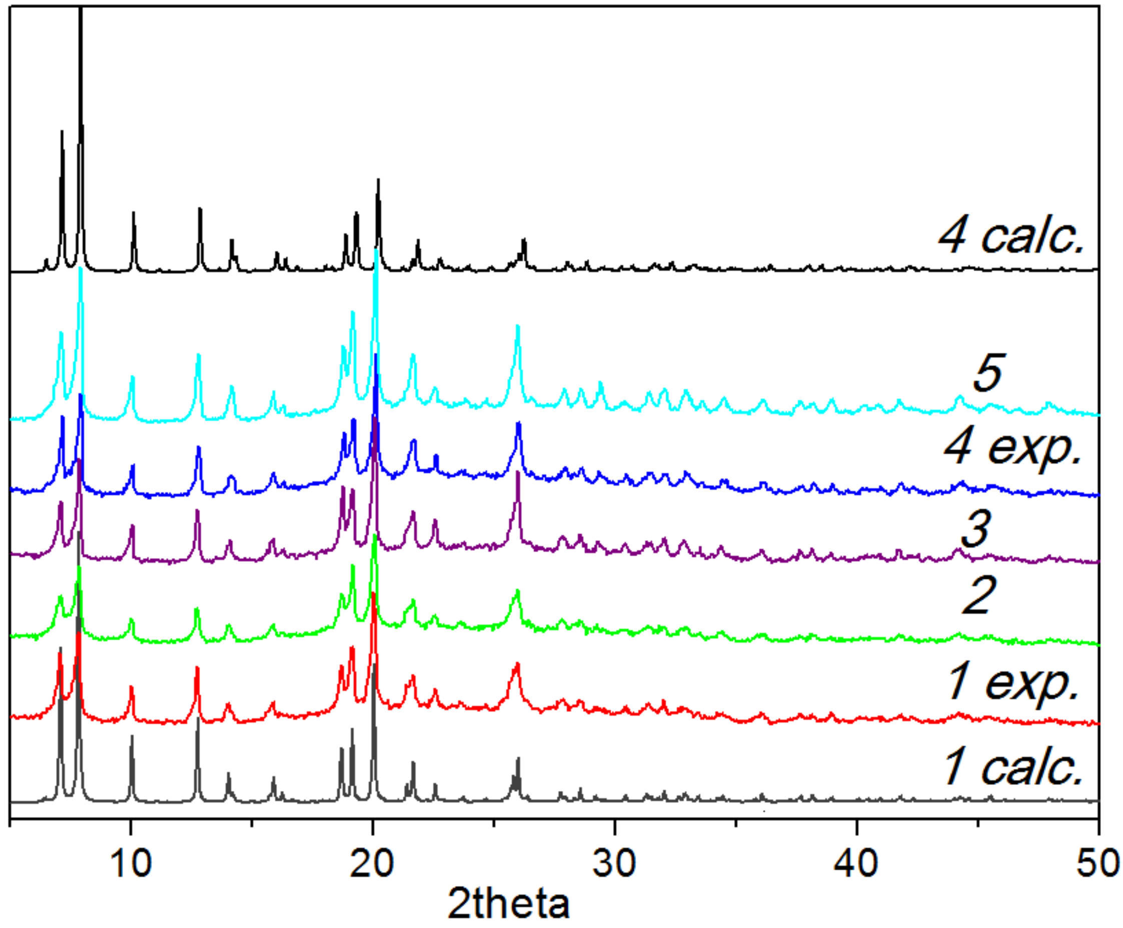

Figure S2. PXRD patterns of the obtained complexes**: 1 – $PPh_4[NdL_4]$, 2 – $PPh_4[SmL_4]$, 3 - $PPh_4[GdL_4]$, 4 - $PPh_4[DyL_4]$, 5 - $PPh_4[TmL_4]$**

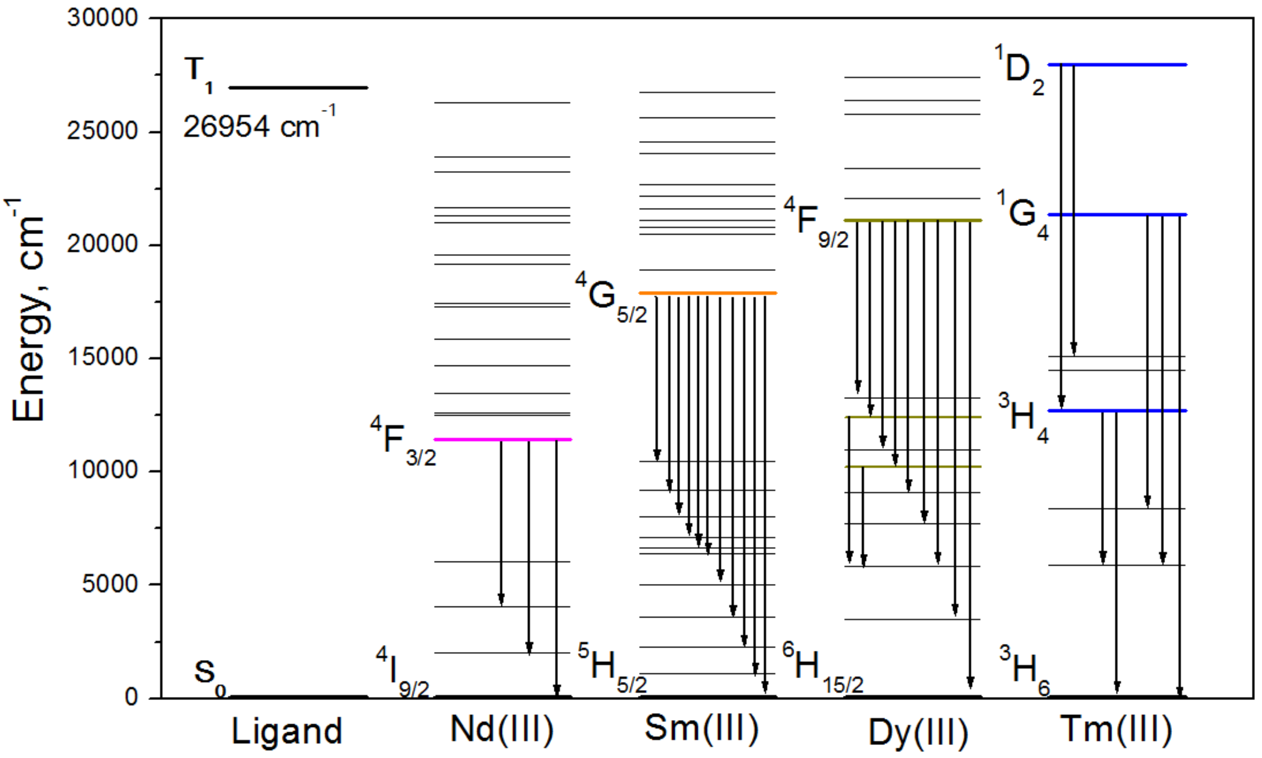

Figure S3. A schematic diagram of the energy levels of the studied lanthanides vs LLTS.

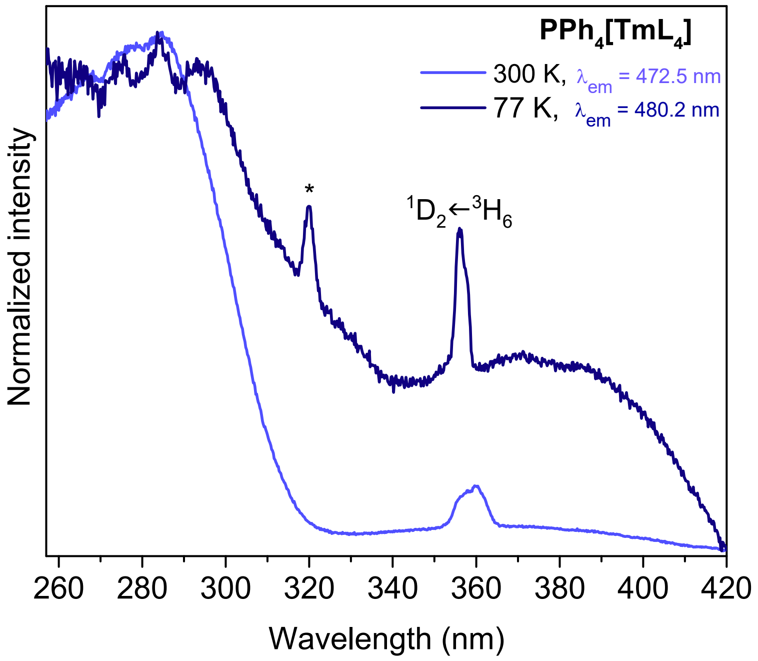

Figure S4. Luminescence excitation spectra of **$PPh_4[TmL_4]$** at 300 and 77 K, λ_{em} = 472.5 nm (300 K), λ_{em} = 480.2 nm (77 K)**.** The band marked with an asterisk appears to be an artifact, in this spectral range the Tm^{III} ion has no absorption band.

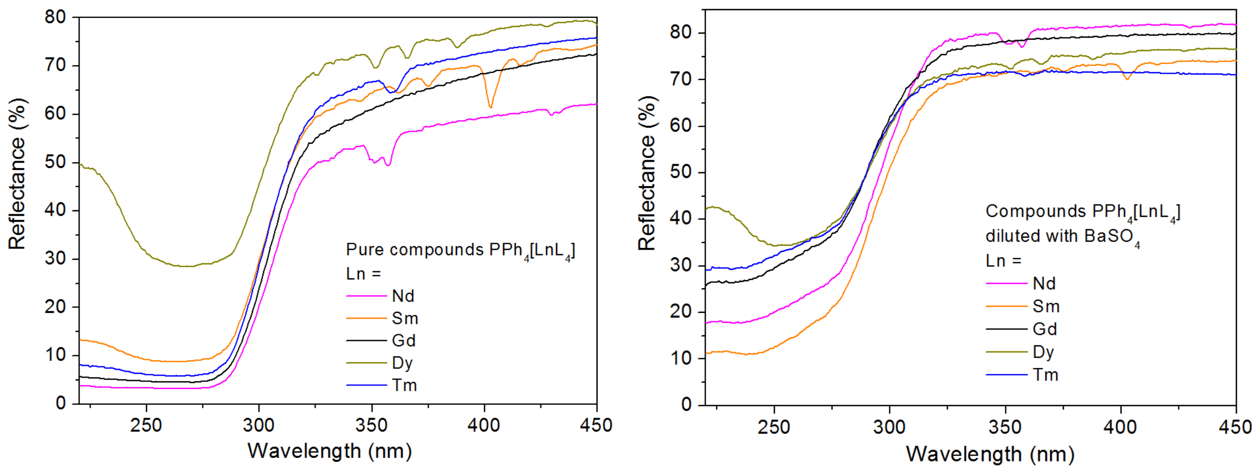

Figure S5. Reflectance spectra of **PPh4[LnL4]** compounds in the solid state undiluted and diluted with $BaSO_4$ at 300 K.

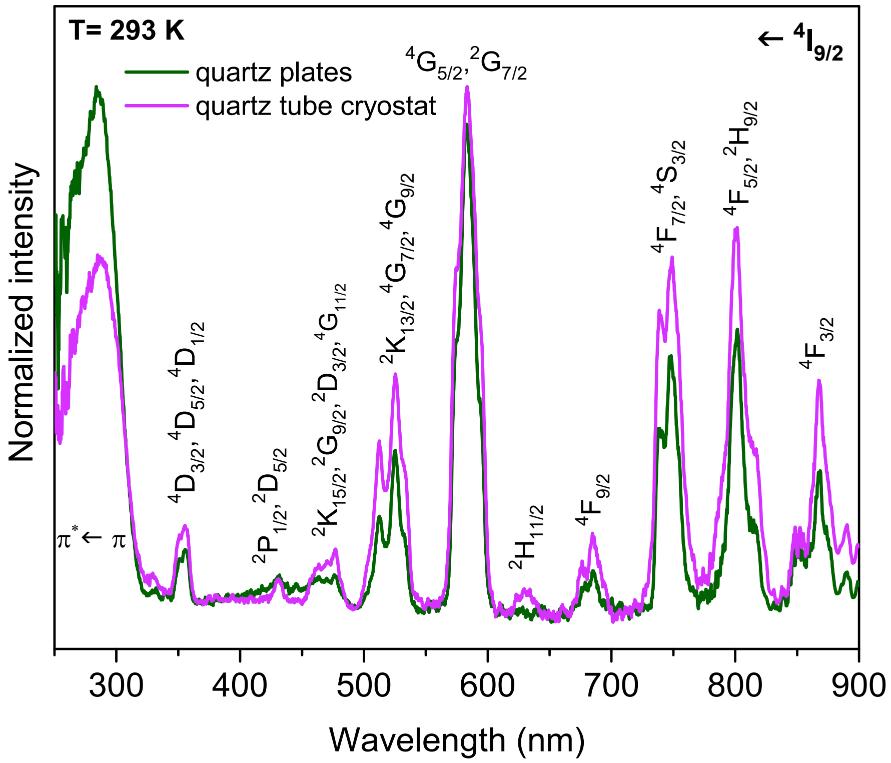

Figure S6. Luminescence excitation spectra of **PPh4[NdL4] at 300 K** obtained for different holders, λ_{em} = 1055 nm.

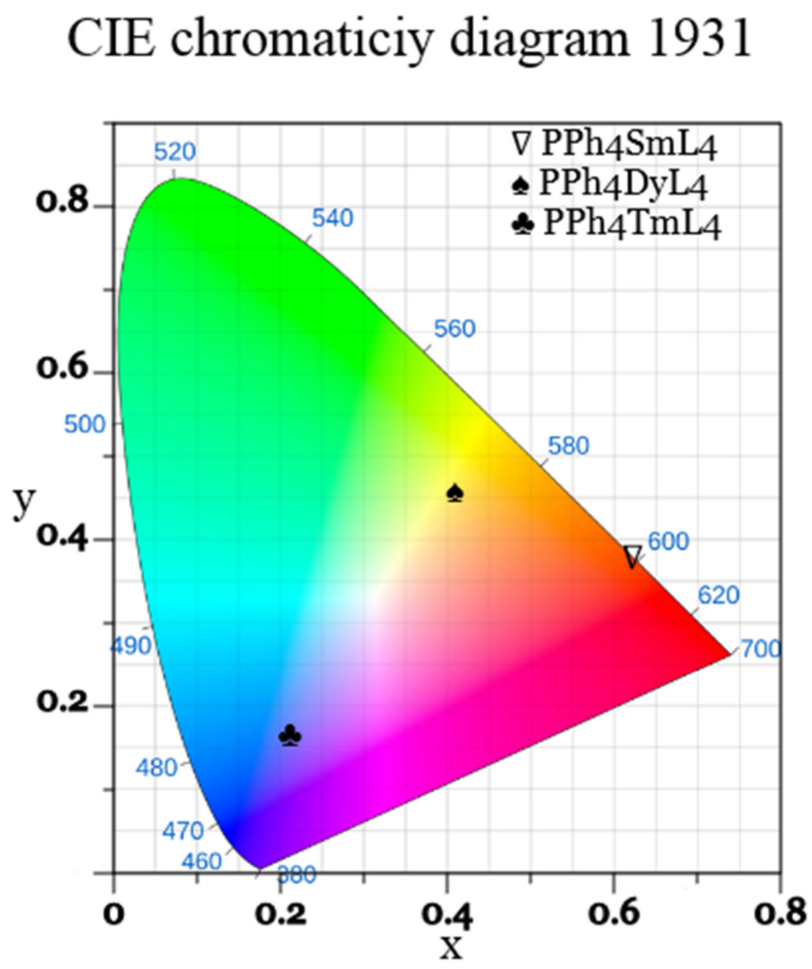

Figure S7. CIE 1931 xy chromaticity diagram for **$PPh_4[SmL_4]$, $PPh_4[DyL_4]$** and **$PPh_4[TmL_4]$** complexes.

a)

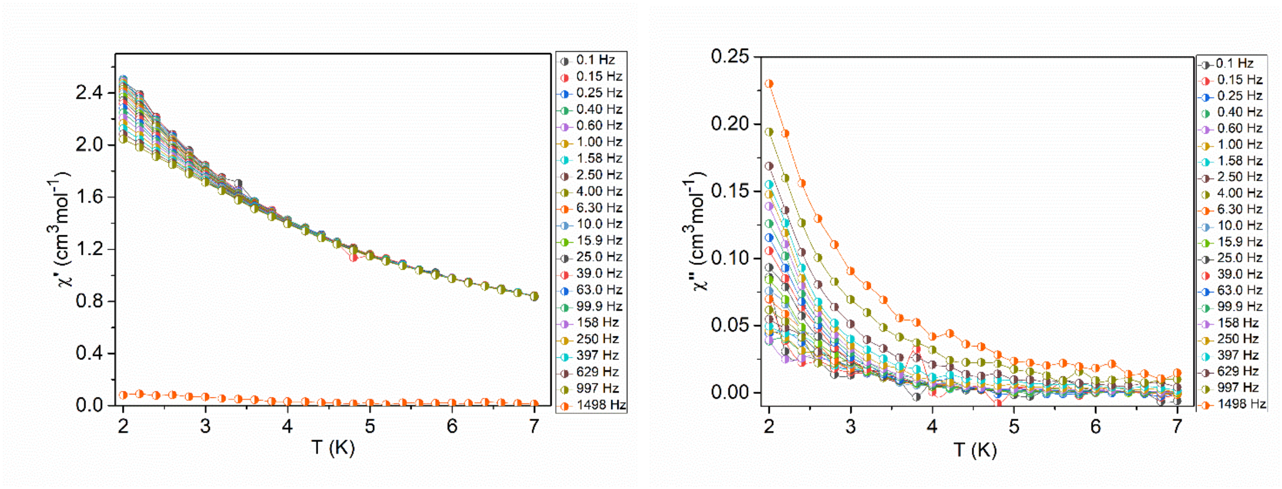

b)

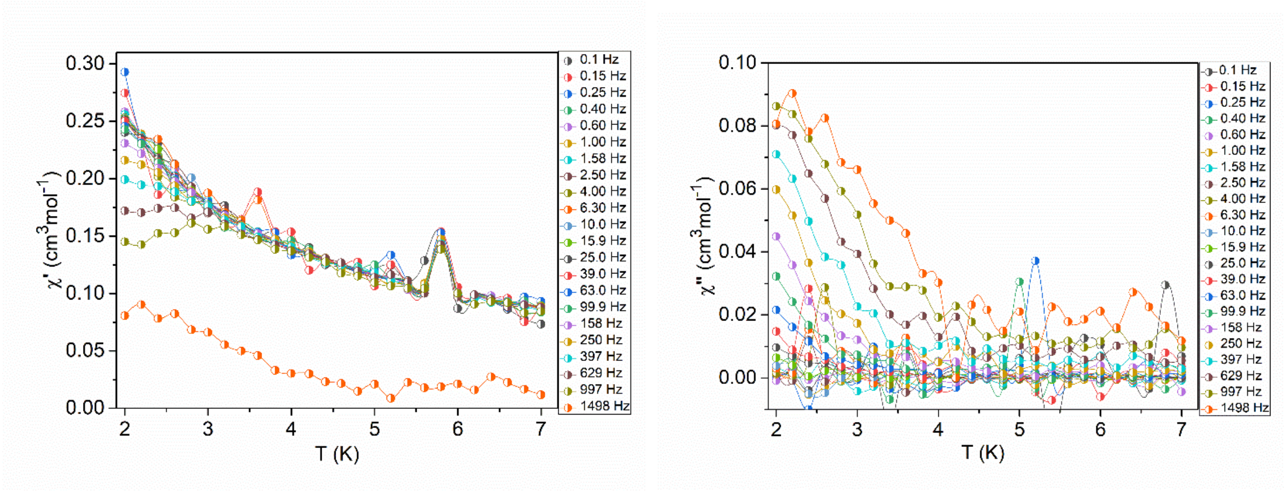

Figure S8. The AC susceptibility data for compounds: a) **$PPh_4[TmL_4]$** and b) **$PPh_4[NdL_4]$** at external DC field: left - temperature dependence of the out-of-phase molar susceptibility; right - temperature dependence of the in-phase molar susceptibility.

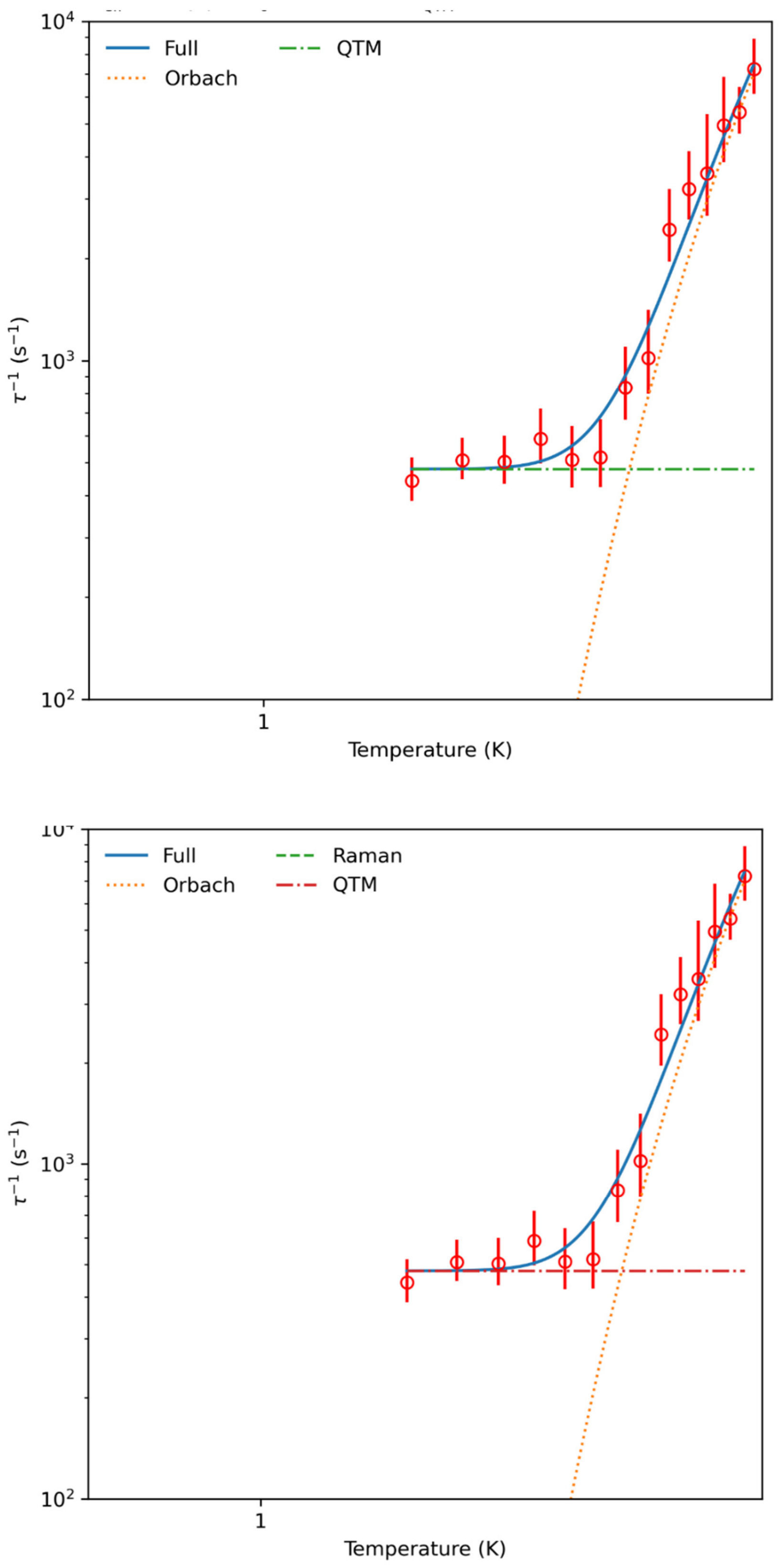

Figure S9. Arrhenius–like plot for **$PPh_4[DyL_4]$.**
